# Targeting BCL-XL in fibrolamellar hepatocellular carcinoma

**DOI:** 10.1172/jci.insight.161820

**Published:** 2022-09-08

**Authors:** Bassem Shebl, Denise Ng, Gadi Lalazar, Carly Rosemore, Tova M. Finkelstein, Rachael D. Migler, Guangrong Zheng, Peiyi Zhang, Caroline S. Jiang, Adam Qureshi, Roger Vaughan, Mark Yarchoan, Ype P. de Jong, Charles M. Rice, Philip Coffino, Michael V. Ortiz, Daohong Zhou, Sanford M. Simon

**Affiliations:** 1 Laboratory of Cellular Biophysics, The Rockefeller University, New York, New York, USA.; 2 Department of Pediatrics, Memorial Sloan Kettering Cancer Center, New York, New York, USA.; 3 The Fibrolamellar Registry, New York, New York, USA.; 4 Department of Medicinal Chemistry, College of Pharmacy, University of Florida, Gainesville, Florida, USA.; 5 Hospital Biostatistics, The Rockefeller University, New York, New York, USA.; 6 The Sidney Kimmel Comprehensive Cancer Center, The Johns Hopkins University School of Medicine, Baltimore, Maryland, USA.; 7 Laboratory of Virology and Infectious Disease, The Rockefeller University, New York, New York, USA.; 8 Division of Gastroenterology and Hepatology, Weill Cornell Medicine, New York, New York, USA.; 9 Department of Biochemistry and Structural Biology and Center for Innovative Drug Discovery, University of Texas Health Science Center at San Antonio, San Antonio, Texas, USA.

**Keywords:** Hepatology, Oncology, Apoptosis inhibitors, Drug therapy, Liver cancer

## Abstract

Fibrolamellar hepatocellular carcinoma (FLC) is a rare and often lethal liver cancer with no proven effective systemic therapy. Inhibition of the antiapoptotic protein BCL-XL was found to synergize with a variety of systemic therapies in vitro using cells dissociated from patient-derived xenografts (PDX) of FLC or cells dissociated directly from surgical patient resections. As BCL-XL is physiologically expressed in platelets, prior efforts to leverage this vulnerability in other cancers have been hampered by severe thrombocytopenia. To overcome this toxicity, we treated FLC models with DT2216, a proteolysis targeting chimera (PROTAC) that directs BCL-XL for degradation via the von Hippel-Lindau (VHL) E3 ligase, which is minimally expressed in platelets. The combination of irinotecan and DT2216 in vitro on cells directly acquired from patients or in vivo using several xenografts derived from patients with FLC demonstrated remarkable synergy and at clinically achievable doses not associated with significant thrombocytopenia.

## Introduction

Fibrolamellar hepatocellular carcinoma (FLC) is a rare and often lethal primary liver cancer of adolescents and young adults (1, 2). Surgical resection is the current standard of care for FLC; however, this is inadequate to cure patients with locally advanced or metastatic disease. There are no proven effective systemic therapies for FLC, although current clinical investigations evaluate the utility of various combinations of chemotherapy, immunotherapy, and targeted therapies (1).

FLC results from a deletion of approximately 400 kB in 1 copy of chromosome 19 that results in the first exon of *DNAJB1*, heat-shock protein 40 (Hsp40), replacing the first exon of *PRKACA*, the catalytic subunit of protein kinase A. The resulting *DNAJB1-PRKACA* is the only recurrent structural rearrangement found in the malignant genome (3–5). Using CRISPR-Cas9 to recreate the approximately 400 kB deletion that generates the fusion chimera was sufficient to recapitulate FLC in mouse models (6, 7). In addition, the direct expression of chimera using a sleeping-beauty transposon produced FLC-like tumors (7), indicating that it is the expression of the chimeric fusion protein, rather than loss of other proteins, that is the oncogenic driver of FLC.

The prevailing approaches for therapeutic treatment of FLC are based on its categorization as a subvariant hepatocellular carcinoma (HCC) (8). However, FLC is distinct from HCC and has a unique pathognomonic molecular driver and distinguishing histopathological features. Furthermore, HCC-directed therapies have not proven to be effective in FLC. Therefore, many investigators have begun to assess oncogenic pathways overexpressed in FLC, including aurora kinase A, EGFR, mTOR, and aromatase (9–11). Unfortunately, approaches targeting these pathways have not proven promising for further investigation (12, 13). In an attempt to find novel effective therapies, we performed an unbiased, high-throughput, small-molecule drug screen. This identified several efficacious compounds that had not been predicted by prior transcriptomic interrogations (14). These included napabucasin (which has several potential mechanisms of action including STAT3 and “stemness” inhibition), panobinostat and fimepinostat (established histone deacetylase [HDAC] inhibitors), irinotecan (a topoisomerase I inhibitor), and navitoclax (an inhibitor of the Bcl-2 family proteins BCL-XL and Bcl-2).

Bcl-2 family proteins have been implicated in cancer progression and resistance to therapy. Many tumors have defects in activation of apoptosis, due to overexpression of Bcl-2 family prosurvival proteins encompassing Bcl-2, BCL-XL, and Mcl-1, among others. Overexpression of BCL-XL has been documented in various tumor types, including multiple solid tumors such as pancreatic ductal adenocarcinoma (15) and HCC (16). In both tumor types, increased BCL-XL was associated with therapeutic resistance and poor prognosis. Thus, apoptotic evasion, a resistance mechanism in many tumors, could be attributed, at least partially, to deregulated expression of BCL-XL.

In FLC, the expression of most members of the Bcl-2 family of proteins was either not significantly altered (Noxa, PUMA, and BID) or was downregulated (Mcl-1, BIM, and Bcl-2), except BAX, BAK, and BCL-XL, whose expression was significantly increased (11, 14). BCL-XL provoked particular interest, as its transcripts were increased by 83% in FLC tumors compared with adjacent normal tissues, and some therapeutically resistant patient-derived xenografts (PDX) tumor lines showed significant upregulation (160%) compared with adjacent normal liver tissue.

Navitoclax, which inhibits both Bcl-2 and BCL-XL, was efficacious against FLC cells dissociated from either PDX or directly from patient tumors, but venetoclax, a specific inhibitor of Bcl-2, had little effect (14). Navitoclax, and other tool compounds that selectively block BCL-XL, synergized with several systemic antitumor agents both in vitro and in vivo against established FLC models. These findings suggest that selective BCL-XL inhibition might lower the apoptotic threshold and result in broad synergy with other agents active against advanced cases of FLC. However, pharmacological inhibition of BCL-XL dramatically reduces the survival of platelets. As a result, the principal dose-limiting toxicity of navitoclax is severe thrombocytopenia, an on-target toxicity that narrows the therapeutic window, particularly in combination with myelosuppressive agents (17–19).

Recently, we reported converting navitoclax into a proteolysis targeting chimera (PROTAC) that can selectively degrade BCL-XL via the VHL ubiquitin E3 ligase (20). Our lead BCL-XL PROTAC, DT2216, has 2 distinct advantages over navitoclax. First, it is significantly less toxic to platelets because platelets express a very low level of the VHL E3 ligase. Second, this particular PROTAC was effective at engaging BCL-XL but not Bcl-2, thus demonstrating greater specificity for BCL-XL than navitoclax. In addition, DT2216, by degrading BCL-XL and recycling, has a longer-lasting effect and an extended pharmacodynamic response. Therefore, targeting BCL-XL with DT2216 has been shown as a promising safe and effective therapeutic strategy in BCL-XL–dependent hematologic malignancies as a monotherapy, as well as in multiple BCL-XL-upregulated solid tumors, when combined with chemotherapeutic agents (20–23). DT2216 is currently undergoing a first-in-human, open-label, multicenter, phase I dose-escalation study with expansion cohorts (NCT04886622).

Here, we demonstrate that DT2216 can synergize with various potential therapeutics for FLC. Strong efficacy against validated FLC models was observed with DT2216 and irinotecan both in vitro and in vivo. DT2216, by itself and in combination with irinotecan, was associated with less thrombocytopenia than standard doses of navitoclax alone, thus establishing its favorable safety profile.

## Results

### In vitro screening for effective therapeutics

From some of the most efficacious classes of agents in a drug-repurposing screen against FLC (14), we selected agents that we felt were most clinically translatable. This set was supplemented with drugs outside of the screen that are currently in clinical use for the treatment of FLC. These agents were first tested for their drug response profile on cells dissociated from 4 independently derived PDX lines. Lines were chosen to include slow- and fast-growing tumors and those that were more drug resistant or drug sensitive. All of them express *DNAJB1-PRKACA* but express variable levels of BCL-XL (Supplemental Figure 1; supplemental material available online with this article; https://doi.org/10.1172/jci.insight.161820DS1). Then, the drugs were assessed in various combinations to quantify synergy.

### SN38 is effective in direct-from-patient FLC tumors

**Figure 1:**
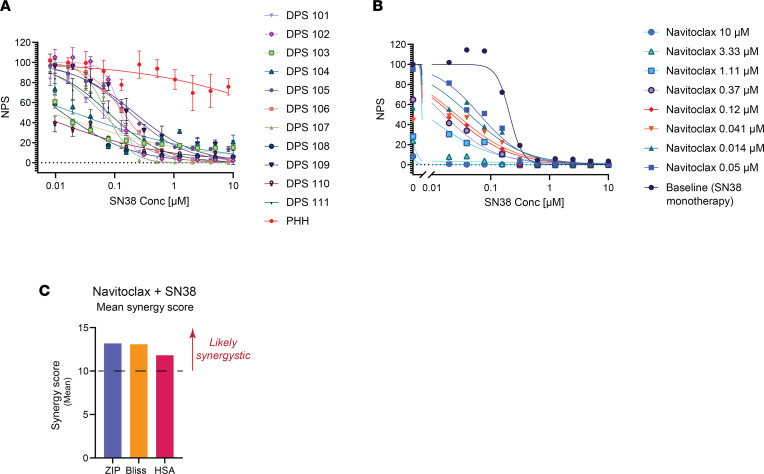
Direct-from-patient screening (DPS) of SN38. (**A**) Dose-response curves of SN38 (TOPO1 inhibitor) against 11 patient samples (samples 101–111) and PHH. Cells were treated at 10 μM–10 nM with 2-fold serial dilution. The *y* axis shows normalized percentage survival calculated as ([positive control – drug response at a given dose]/[positive control – negative control]) × 100. The *x* axis shows the concentration in μM. Data are presented as mean ± SD (*n* = 3). (**B**) Dose-response curves of SN38 in the presence of increasing concentrations of navitoclax. Synergy screening was done on patient sample DPS 102. (**C**) Comparison of synergy models for SN38-navitoclax combo from **B**. NPS, normalized percentage survival.

One of the highest-ranked targets from the prior drug screen was SN38, the active metabolite of irinotecan, a topoisomerase I (TOPO1) inhibitor. Given its rank in the drug screen and broad oncologic utility, we tested SN38 against 11 different FLC tumors freshly resected from patients. A clear difference was consistently observed relative to normal primary human hepatocytes (PHH) (Figure 1A).

While SN38 ranked highly in our previous screen, it showed a variable response against independently derived PDX (14). We had previously noted that Navitoclax, a dual Bcl-2/BCL-XL inhibitor was a top hit in our previous screen. Furthermore, BCL-XL overexpression was associated with therapeutic resistance. Specifically, blocking BCL-XL using A1331852 showed an enhanced response when combined with some of our top hits — HDAC inhibitors and TOPO1 inhibitors. In contrast, blocking Bcl-2 using venetoclax (ABT199), or Mcl-1 using AZD5991, showed modest to no synergistic effect. This suggested that BCL-XL is primarily responsible for FLC drug resistance.

We explored whether inhibiting BCL-XL would enhance the antitumor response of SN38 on ex vivo cells derived from an FLC tumor immediately after resection from the patient. By inhibiting BCL-XL using navitoclax, we demonstrated progressive augmentation of the response to irinotecan (Figure 1B), which was found to be synergistic (Figure 1C).

### Other clinical agents

#### HDAC inhibitors.

HDACs are upregulated in multiple tumors, and inhibiting HDACs to modulate transcription is an emerging therapeutic modality in many cancers. Based on our previous screens, we chose 2 clinical-stage HDAC inhibitors for further testing. Panobinostat is an oral pan-HDAC inhibitor and the first HDAC inhibitor approved for treating multiple myeloma. Panobinostat was effective against PDX from primary tumors (PDX 31, efficacy at 1 μM [E_1_] = 87% and EC_50_ = 14.7 nM) and metastatic tumors (PDX 34, E_1_ = 98% and EC_50_ = 12.3 nM), although it was not as efficacious against our more therapeutically resistant lines (PDX 32 and PDX 33). Panobinostat showed limited toxicity against the PHH control (E_1_ = 36% and EC_50_ > 10 μM) (Supplemental Figure 2A). Fimepinostat (also known as CUDC-907) is a dual inhibitor against HDACs and phosphotidyl-inositol 3 kinase (PI3K), which has been in clinical trials for treatment of lymphoma, breast cancer, multiple myeloma, and NUT midline carcinoma and is currently in a trial for children and young adults with solid tumors (NCT02909777; clinicaltrials.gov). PDX of metastatic-origin FLCs showed a mixed response (PDX 34, E_1_ = 99% and EC_50_ in the single-digit nanomolar range), with the more resistant tumors showing less responsiveness (PDX 32 and 33, E_1_ = 10%–13%). Compared with panobinostat, fimepinostat was effective, yet to a lesser extent, against the primary FLC PDX (PDX 31, E_1_ = 68% and EC_50_ = 570 nM) (Supplemental Figure 2B).

#### MEK inhibitors.

Mitogen-activated extracellular signal–regulated kinase (MEK) is in the MAPK signaling pathway that has been implicated in multiple cancers. Trametinib is an allosteric inhibitor of MEK and has been approved for treating metastatic melanoma and has been proposed as a therapeutic for FLC based on its effects on mouse cells expressing *DNAJB1-PRKACA* and overexpressing TGF-α (24). However, trametinib failed to show any significant responses against either the primary tumor (PDX 31, E_1_ = 38% and EC_50_ > 10 μM) or the metastatic lines of FLC PDX (PDX 32, E_1_ = 9%; PDX 33, E_1_ = 11.5%; and PDX 34, E_1_ = 21%) (Supplemental Figure 2C), consistent with our previous tests (14).

#### Chemotherapeutic agents.

We tested chemotherapeutic agents that are being used to treat FLC in the clinic, including cisplatin, gemcitabine (as a single agent or in combination with oxaliplatin such as GEMOX), fluorouracil, paclitaxel, temozolomide, and vincristine (Supplemental Figure 2, D–J). Despite being used in multiple clinical trials currently, these chemotherapeutic agents showed a variable and nonconsistent response against different FLC PDX lines, usually at higher doses that would be expected to be clinically achievable and with a limited therapeutic window.

#### Kinase inhibitors.

An enzymatically active DNAJB1-PRKACA kinase is critical for almost all cases of FLC (7). Uprosertib (GSK2141795) is in clinical trials as an AKT inhibitor with an IC_50_ of 489 nM and 1,039 nM for AKT1 and AKT2, respectively, but it also inhibits PRKACA with an IC_50_ of 21 nM (25). Uprosertib showed efficacy in our previous drug screens (14). It showed a variable response against different FLC PDX but was always more efficacious against FLC, whether from primary tumors (PDX 31, E_1_ = 68% and EC_50_ = 253 nM) or metastases (PDX 32, E_1_ = 26% and EC_50_ = 1.9 μM; PDX 33, E_1_ = 42% and EC_50_ = 1.5 μM; and PDX 34, E_1_ = 50% and EC_50_ =801 nM), than PHH (Supplemental Figure 2K).

#### mTOR kinase inhibitors.

Everolimus is a derivative of Rapamycin and a specific inhibitor of mammalian target of rapamycin 1 (mTOR1), which has been tested in combination with an aromatase inhibitor for FLC (13). Everolimus showed no response against all PDX tested (Supplemental Figure 2L).

#### Tyrosine kinase inhibitors.

Receptor tyrosine kinases (RTKs) are involved in growth and metastasis of many cancers, and their inhibitors are a wide family of clinically established antineoplastic agents. Tyrosine kinase inhibitors showed a variable response against the primary and metastatic lines of FLC PDX (Supplemental Figure 2, M–P). Neratinib, which interestingly was not as active clinically as monotherapy but had a reported partial response in combination with checkpoint inhibition (12), showed the best response in the primary line (PDX 31, E_1_ = 46% and EC_50_ = 1.3 μM) (Supplemental Figure 2O).

Three categories of drugs showed consistent efficacy (E_1_ > 50%) against more than 1 FLC line: HDAC inhibitors (panobinostat and fimepinostat), TOPO1 inhibitors (irinotecan, represented by its active metabolite SN38), and a PKA kinase inhibitor (uprosertib) (Supplemental Figure 2R). We chose irinotecan for further testing because it has been extensively tested in the clinic and showed a robust response in the screening of FLC cells directly from patients.

### PROTAC-mediated BCL-XL degradation in FLC

The level of BCL-XL protein (Supplemental Figure 1D), or BCL-XL transcript (14), varied among different PDX lines but showed no correlation with the aggressiveness of the tumor, with the origin of the tumor (primary or metastases), or with the relative expression level of oncogenic kinase DNAJB1-PRKACA. However, the level of BCL-XL correlates with the therapeutic resistance of the different FLC tumor lines, especially to HDAC inhibitors and TOPO1 inhibitors (14). PDX 32 is our most therapeutically resistant line, and it had 4.5-fold the BCL-XL level of PDX 31, our most sensitive line. PDX 33, the next most resistance line, had twice the BCL-XL protein level of PDX 31. The PDX 31 (1-fold) and 34 (0.8-fold) showed relatively similar values for BCL-XL (Supplemental Figures 1 and 2).

In tests of cells freshly dissociated from the PDX, blocking BCL-XL with navitoclax was efficacious both as monotherapy and in drug combinations against FLC models. Given the aforementioned concerns about the clinical viability of combination studies including navitoclax, due to severe thrombocytopenia, we next tested an alternative agent for reduction of BCL-XL. DT2216, a PROTAC in clinical studies, is a BCL-XL degrader that selectively targets an E3 ligase, von Hippel-Lindau (VHL). Because VHL ligase is minimally expressed in platelets, DT2216 circumvents the dose-limiting toxicity associated with navitoclax (20). DT2216, as a single agent, showed an effective response (E_1_ = 53%) against cells dissociated from PDX 31, a weaker response against PDX 32 (E_1_ = 16%) and PDX 33 (E_1_ = 31%), and it showed no efficacy against PDX 34 and no toxic effect against PHH (Supplemental Figure 2Q).

**Figure 2:**
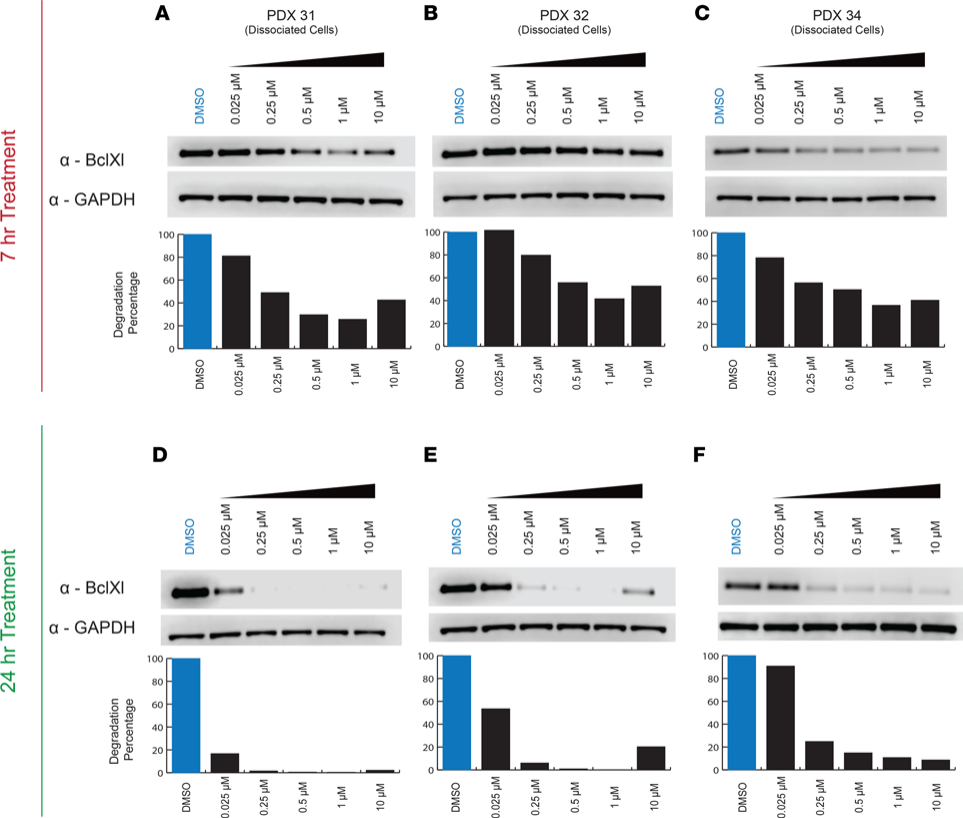
Immunoblot analysis of BCL-XL in dissociated cells from FLC PDX. (**A**–**F**) Cell lysates were blotted after treatment with indicated concentrations of DT2216 for 7 hours (**A**–**C**) or 24 hours (**D**–**F**). GAPDH was used as a loading control for all immunoblot analysis presented. Data were corrected with a normalization factor against GAPDH and are presented as a percentage of the DMSO treated cells (control). The upper panel shows the immunoblots, and the lower panel shows the densiometric analysis performed using LI-COR.

When we treated cells with DT2216, the BCL-XL level declined in all of the FLC lines tested (Figure 2). The effect was concentration dependent and was apparent after 7 hours of treatment and greater at 24 hours. The PDX that were more sensitive to DT2216 (PDX 31 and PDX 34) showed more degradation at 7 hours than the nonresponsive PDX 32 (Figure 2, A–C). At 24 hours, BCL-XL was reduced to almost undetectable levels at 0.25 μM in all 3 lines. In 1 line (PDX 32), the 10 μM dose was less efficacious then lower doses in reducing BCL-XL levels, suggestive of the hook effect observed with some PROTACs (26) (Figure 2, D–F).

### Synergy screening

We explored the potential for synergy between degradation of BCL-XL and inhibition of other targets using SynergyFinder 2.0. We evaluated the results based on (a) a quantitative assessment using 3 different synergy models (zero interaction potency [ZIP], Bliss, and highest single agent [HSA]) (27), (b) a critical evaluation of the dose-response curves to look for synergistic potency and/or synergistic efficacy, and (c) identification of biologically and clinically relevant interactions and therapeutic efficacy.

**Figure 3:**
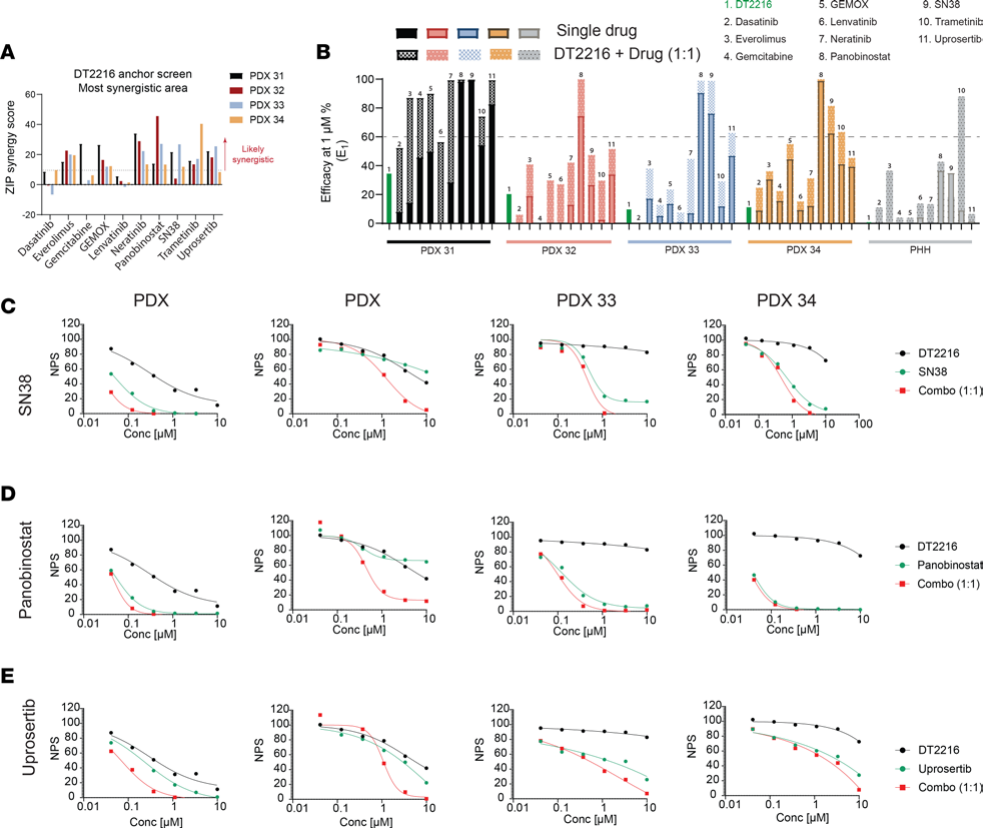
DT2216 Anchor Screen. (**A**) The most synergistic area scores (synergy score for the most synergistic 2 × 2 dose region) out of the matrix for each drug combination, (**B**) The efficacy of selected drugs as single agents or in combination with DT2216 at a ratio of 1:1 at 1 μM. Dose-response curves of selected DT2216 combinations. (**C**–**E**) Dose-response curves of selected drugs tested against 4 FLC PDX lines and a PHH control. Drugs are plotted either as single agents or in combination with DT2216 in a ratio of 1:1. Drugs were tested at 10 μM–41 nM with 3-fold serial dilution. The *y* axis shows normalized percentage survival calculated as ([positive control – drug response at a given dose]/[positive control – negative control]) × 100. The *x* axis shows the concentration in μM. Topoisomerase I inhibitors (SN38) (**C**), HDAC inhibitors (panobinostat) (**D**), and Kinase inhibitors (uprosertib) (**E**). NPS, normalized percentage survival.

Synergy was evaluated using a DT2216 anchor screen against other agents using a 6 × 6 dose-response matrix from 41 nM to10 μM, and the ZIP scores were plotted (Figure 3A and Supplemental Figures 3–6). In all 4 FLC PDX lines, synergism was detected between DT2216 and a number of different agents. However, a drug combination with a synergistic effect is not always reflective of its therapeutic applicability or therapeutically meaningful efficacy. Thus, we compared the therapeutic efficiency for all 4 PDX lines tested as single agents and in the presence of DT2216. We defined therapeutic efficacy as > 60% cell death. In the presence of DT2216, the HDAC inhibitors panobinostat and fimepinostat and the TOPO1 inhibitor SN38 showed the highest efficacy, followed closely by the kinase inhibitor uprosertib. This was observed for all PDX lines, including line 32, which was the most resistant to other therapeutics (Figure 3, B–E). Other selected drugs showed minimal to no effect at < 1 μM and/or no specific efficacy against FLC. Panobinostat showed the highest degree of synergy with DT2216. This is consistent with our previous results screening panobinostat and navitoclax against FLC both on dissociated cells and in preclinical models (14).

### Assessment of DT2216 in vivo

We initially tested the combination of irinotecan and navitoclax and found that the combination had a therapeutic benefit in FLC (Supplemental Figure 7). Unfortunately, the groups that received navitoclax were not in good health for further rounds of treatment due to navitoclax-induced thrombocytopenia, limiting the ability to meaningfully advance this approach clinically.

**Figure 4:**
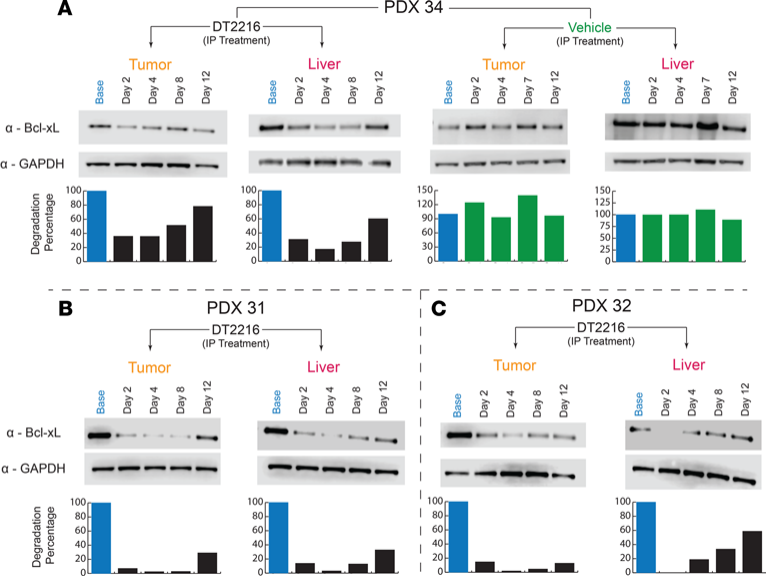
DT2216 induces degradation in vivo in FLC PDX. PDX mice were treated with a single dose of the i.p. formulation, and tumor tissue was collected from the tumor and liver of each mouse. Each time point represents an independent mouse. BCL-XL level was monitored using Western blotting. GAPDH was used as a loading control for all immunoblot analysis presented. Data were corrected with a normalization factor against GAPDH and are presented as a percentage of the vehicle-treated (Base) cells as a control. The upper panel shows the immunoblots, and the lower panel shows the densiometric analysis performed using LI-COR. (**A**) PDX 34 treated with the i.p. formulation in tumor and liver samples, along with the vehicle control. (**B**) PDX 31 treated with the i.p. formulation in tumor and liver samples. (**C**) PDX 32 treated with the i.p. formulation in tumor and liver samples.

In light of this promising activity of navitoclax in combination with irinotecan for FLC models, we next assessed whether DT2216 might have comparable or improved targeting of BCL-XL but with a lower degree of thrombocytopenia and, therefore, a wider therapeutic window. The efficacy of degrading BCL-XL was tested with a single injection of DT2216, and the levels of BCL-XL were assessed both in s.c. tumor and in the mouse liver. Both the i.v. and i.p. formulations of DT2216 were effective at degrading BCL-XL in PDX 34 (Figure 4A and Supplemental Figure 8) and in PDX 31 and PDX 32 (Figure 4, B and C). We noticed a rapid degradation of BCL-XL by the second day and then observed its slow but gradual recovery over the course of the experiment in all 3 PDX lines tested. Given the time course of degradation, we explored 2 different DT2216 dosing strategies, either once or twice a week; the former was chosen as reasonable based on the time course of BCL-XL depletion in FLC, and the latter was the approach used in the current phase I clinical study.

To compare the in vivo safety of DT2216 to navitoclax*,* naive NOD/SCID-γ (NSG) mice, were randomized into 4 treatment groups: vehicle, navitoclax (50 mg/kg/qd/p.o.), DT2216 (15 mg/kg/1 time only/i.p.), and DT2216 (15 mg/kg/1 time only/i.v.). In our combination studies, we treated mice with navitoclax daily and with DT2216 as a single dose, as it has been shown to significantly reduce BCL-XL levels in tumor xenografts for more than a week (20). Blood was collected from mice on day 4, when platelet counts remain at the lowest levels after single DT2216 treatment (20). Navitoclax-treated mice had ~97% reduction in blood platelet count as compared with vehicle-treated controls, whereas platelets of DT2216-treated mice were reduced 42% (i.v. formulation) and 61% (i.p. formulations), which is in agreement with our previous report (20). There was no discernable effect on WBCs, RBCs, or hemoglobin (Supplemental Figure 9). The results are in agreement with previous work, wherein the nadir of blood platelets occurred within 6 hours of navitoclax administration, with a rapid rebound and normalization at 3 days after treatment. DT2216, instead, induces a more delayed and moderate platelet reduction with a nadir approximately 3 days after treatment (20).

For further validation of the safety of the treatment approach in vivo, we obtained a complete blood count and hepatic function test in NSG mice (Supplemental Figure 10). Mice were randomized into 3 treatment groups: vehicle, irinotecan (5 mg/kg), and DT2216 (15 mg/kg/i.v.)/irinotecan (5 mg/kg) combo. DT2216 was administrated twice a week throughout the entire treatment cycle. Irinotecan was administered 5 days per week for 2 consecutive weeks, with the third/last week of the cycle being off treatment. Blood and liver samples were collected from treated mice after 2 weeks of the treatment cycle. For all groups, the platelet count remained in the normal range of NOD-SCID mice (651–2,055 K/μL), but there was a slight elevation of the platelets in the combo treatment group as compared with the vehicle control (Supplemental Figure 10). Liver function tests showed that, for all groups, both ALT and total bilirubin (TBIL) were within the normal range (27–195 U/L for ALT and 0.2–0.6 mg/dL for TBIL, respectively).

### In vivo test of combining irinotecan and DT2216

**Figure 5:**
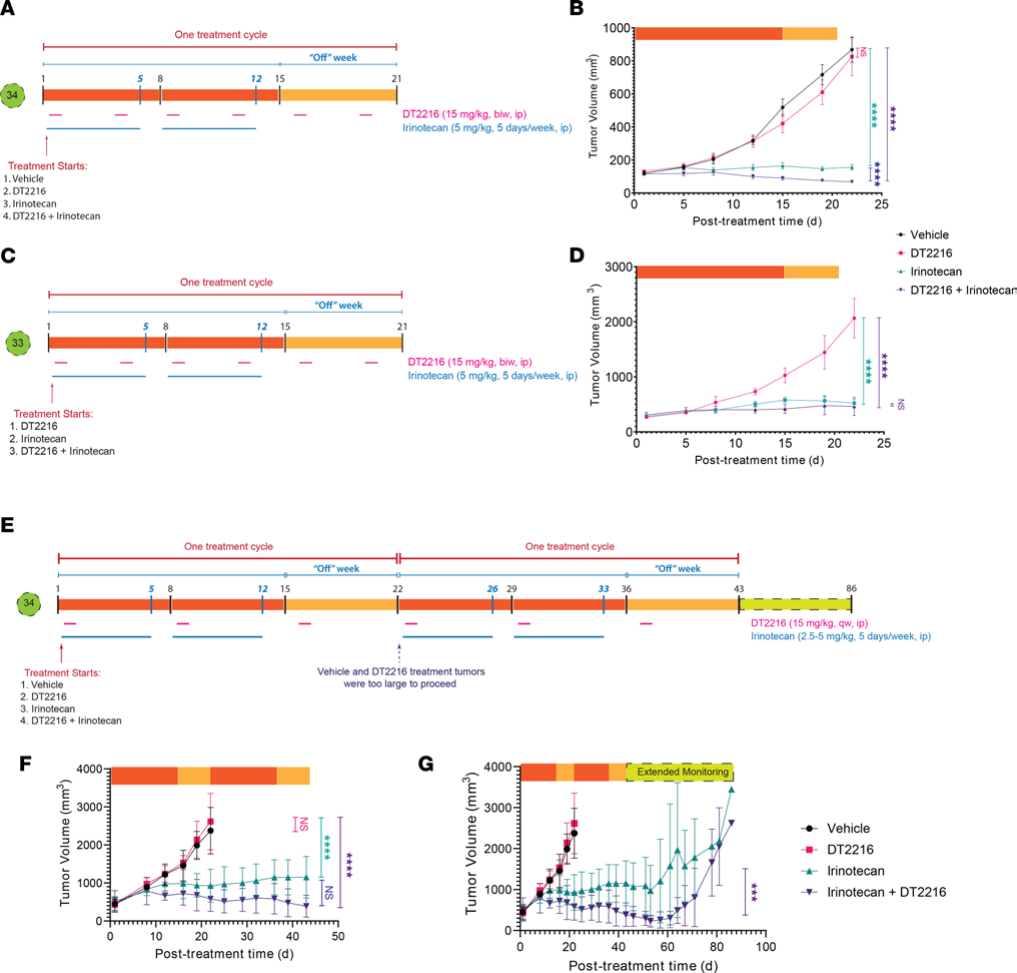
DTT216 administered i.p. twice a week combined with irinotecan. (**A** and **C**) Illustration of treatment timeline for PDX 34 and PDX 33. DT2216 was administered i.p. twice a week for the entire treatment cycle; irinotecan was administered for 5 days a week for 2 weeks. The third week, the mice were off the treatment with irinotecan. (**B** and **D**) Changes in tumor volume over the course of treatment. Data are presented as the mean ± SEM (*n* = 7 for vehicle and irinotecan treatment groups, *n* = 6 for DT2216, *n* = 9 for other treatment groups at the start of the treatment for PDX 34, *n* = 3 for DT2216 and irinotecan, and *n* = 4 for the combo treatment at the start of treatment for PDX 33). ****P < 0.0001 in indicated comparisons. DT2216 with 2 cycles of treatment. (**E**) Illustration of treatment timeline for PDX 34. DT2216 was administered i.p. twice a week for the entire treatment cycle. A low dose of irinotecan (2.5 mg/kg) was administered i.p. for 5 days a week for the first treatment cycle, followed by an intermediate dose of irinotecan (5 mg/kg) administered for the second treatment cycle. The third week of each treatment cycle, the mice were off irinotecan. (**F**) Changes in tumor volume over the course of the treatment cycle. Data are presented as the mean ± SEM (*n* = 4 for all treatment groups at the start of the treatment for PDX 34). (**G**) Changes in tumor volume over the entire timeline (treatment cycle + extended monitoring beyond treatment). ***P < 0.001 in indicated comparisons, as determined by Linear mixed-effects regression model.

The mice were treated twice weekly with DT2216 (15 mg/kg) i.p. throughout the treatment cycle, while irinotecan (5 mg/kg) was given i.p. 5 days a week for 2 consecutive weeks and discontinued in the last week. Mice were randomized into 3 (PDX 33) or 4 (PDX 34) treatment groups and treated for 1 cycle (Figure 5A). In PDX 34, the DT2216 monotherapy showed modified progressive disease (mPD) with a mean tumor growth inhibition (TGI) of 7%; irinotecan showed modified stable disease (mSD) (TGI of 95%), and DT2216-irinotecan combo treatment showed modified partial response (mPR) (TGI of 106%) (Figure 5B). In PDX 33, a more resistant PDX line, we observed a similar trend, except both therapies resulted in stable disease. With irinotecan monotherapy, there was an mSD of TGI 82%, and for the combo therapy, there was an mSD of TGI 88% (Figure 5, C and D). Mice remained active and alert during the course of treatment with no significant weight loss (<20%).

The improved tolerability of DT2216 relative to navitoclax allowed us to explore multiple treatment cycles. We randomized PDX 34 mice into 4 treatment groups: vehicle, DT2216, irinotecan, and a combination of DT2216 and irinotecan. We administered DT2216 (15 mg/kg) once a week by i.p. injection throughout the treatment cycle and irinotecan 5 days a week for the first 2 consecutive weeks of each 3-week treatment cycle. For the first treatment cycle, irinotecan was given at a low dose (2.5 mg/kg); after demonstrating tolerability, we increased it, for the following cycles, to an intermediate dose (5 mg/kg) (Figure 5E).

The vehicle and DT2216 treatment groups were discontinued after 1 treatment cycle, as the tumors had grown to the limit allowed in our protocol (Figure 5F). At the end of 1 treatment cycle, the TGI of the irinotecan group was 74%, and the combo group showed great efficacy at 90% TGI. Through the second cycle, the irinotecan group showed mSD, while the combination was more effective with mPR. After the second cycle, we stopped all treatment for 6 weeks. The irinotecan group relapsed with mPD detectable by 55 days, 12 days after terminating treatment, while the combination did not show mPD until 21 days after terminating treatment (Figure 5G). Thus, efficacy was seen after 1 round of therapy, even in our most rapidly growing model for FLC.

FLC is a cancer that is cared for by both pediatric and medical oncologists. Although irinotecan is administered in both pediatric and medical oncology contexts, pediatric regimens tend to utilize lower doses and more protracted treatment with irinotecan, whereas adult oncology regimens tend to have higher doses at less-frequent intervals. This has practical and toxicity implications that are well documented, but we were interested in exploring which approach might impact the efficacy of irinotecan in FLC models. As a result, mice were randomized into 4 treatment groups: vehicle, a single high irinotecan dose (25 mg/kg) at the start of the cycle, an intermediate irinotecan dose (5 mg/kg) given 5 days a week i.p. for 2 consecutive weeks of the 3-week treatment cycle, and the intermediate irinotecan dose in combination with DT2216 (15 mg/kg) given twice a week throughout the treatment cycle. Mice were treated for 1 cycle only and then monitored for an extended period (Supplemental Figure 11A). At the end of the cycle, the high irinotecan showed progressive disease with a TGI of 47%, and the intermediate irinotecan dose had a slightly progressive disease with a TGI of 82%. Only the combination of intermediate irinotecan dosing with DT2216 showed stable disease with a TGI of 97%. After the end of treatment, the high-irinotecan group demonstrated progressive disease. The intermediate irinotecan group and the combination had mSD for 2 weeks and then showed mPD (Supplemental Figure 11, B and C). Based on these results, we pursued a more-frequent, lower-dosing regimen of irinotecan, consistent with data supporting a more effective protracted course of irinotecan (28).

Efficacy of the clinical IV formulation of DT2216

**Figure 6:**
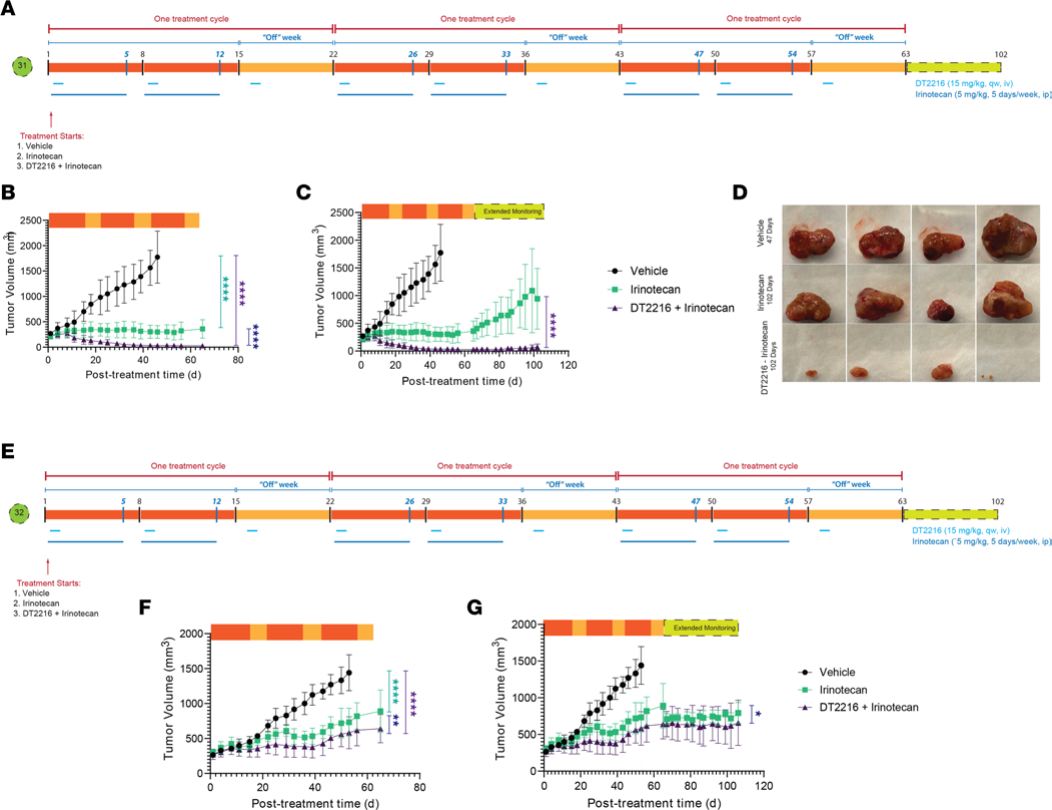
The i.v. formulation of DT2216 synergizes with irinotecan, leading to a sustained complete remission in FLC PDX. (**A**) Illustration of treatment timeline for PDX 31. DT2216 was administered i.v. once a week for the entire treatment cycle; an intermediate dose of irinotecan (5 mg/kg) was administered for 2 treatment cycles, 5 days a week. The third week of each treatment cycle, the mice were off irinotecan. (**B**) Changes in tumor volume over the course of the treatment cycle. Data are presented as the mean ± SEM (*n* = 4 for vehicle group and *n* = 5 for the rest of the treatment groups at the start of the treatment for PDX 31). (**C**) Changes in tumor volume over the entire timeline (treatment cycle + extended monitoring beyond treatment). (**D**) Tumor images of FLC PDX–engrafted mice after the end of treatment and monitoring at day 47 for vehicle and day 102 for the irinotecan and irinotecan + DT2216 cohorts. ****P < 0.0001 in indicated comparisons. Resistant FLC models exhibit durable clinical benefit to DT2216 and irinotecan. (**E**) Illustration of treatment timeline for PDX 32. DT2216 was administered i.v. once a week for the entire treatment cycle; an intermediate dose of irinotecan (5 mg/kg) was administered for 2 treatment cycles, 5 days a week. The third week of each treatment cycle, the mice were off irinotecan. (**F**) Changes in tumor volume over the course of the treatment cycle. Data are presented as the mean ± SEM (*n* = 5 for vehicle group, *n* = 6 for the irinotecan treatment group, and *n* = 7 for the combo treatment group at the start of the treatment for PDX 32). (**G**) Changes in tumor volume over the entire timeline (treatment cycle + extended monitoring beyond treatment). *P < 0. 05, **P < 0.01, and ****P < 0.0001, as determined by Linear mixed-effects regression model.

These initial studies were performed with an experimental i.p. formulation, as has been used in prior preclinical studies, but the i.v. formulation of DT2216 provides a clinically viable option and is the formulation used on the current phase I study. To test the efficacy of the i.v. formulated DT2216, mice were randomized into 3 treatment groups: vehicle only, irinotecan (5 mg/kg) given 5 days a week for the first 2 consecutive weeks of each treatment cycle, and a third group where the same dose of irinotecan was used but with the addition of DT2216 (15 mg/kg) delivered through tail vein injection throughout the cycle (Figure 6A). This protocol was tested on PDX 31, which is typical of most of our PDX for growth and drug sensitivity. At the end of the second cycle, the tumors in the vehicle control had to be sacrificed according to our protocol. At this point, the irinotecan group showed stable disease with a TGI of 95% and the combo showed a complete response with a TGI of 111% (Figure 6B). The efficacy of the irinotecan and the combination continued through the third cycle (Figure 6C).

During extended monitoring, after the end of treatment, only the irinotecan group developed progressive disease. Strikingly, in combination with DT2216, the complete response continued throughout the 6 weeks of the monitoring period (Figure 6, B and C). Mice were sacrificed, and tumors were inspected by the end of the monitoring period, demonstrating a substantial treatment effect in the combination arm (Figure 6D). The protocol was repeated with PDX 32, our most drug-resistant PDX line (Figure 6, E–G). While a reduction of tumor growth was observed with irinotecan, and a statistically greater reduction was observed with the combination, both groups showed slow progressive growth of the tumor during the treatment, which then remained stable during the monitoring period.

### Clinical utility of irinotecan in advanced FLC

**Figure 7:**
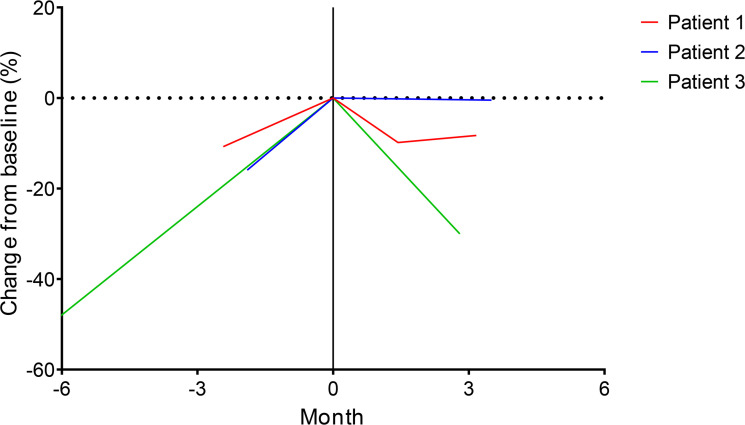
Irinotecan clinical activity in patients with advanced FLC. Change in tumor target lesions by RECIST 1.1 is shown before and after commencement of irinotecan-based systemic therapy for 3 patients identified through the Fibrolamellar Registry. All 3 patients had tumor progression prior to starting irinotecan in month 0. Patient 1 received irinotecan monotherapy, whereas patients 2 and 3 received irinotecan in combination with other systemic therapies. By RECIST 1.1, patients 1 and 2 achieved stable disease as a best response to therapy, and patient 3 had an unconfirmed partial response.

We examined a patient-run medical registry, the Fibrolamellar Registry (www.fibroregistry.org), for patients with FLC who had received agents included in this investigation, and we identified 3 patients with FLC who had received irinotecan in clinical practice (although no other TOPO1 nor BCL-XL targeting agents were reported). All 3 patients had received debulking surgeries and multiple lines of systemic therapy in the metastatic setting. Prior to receiving irinotecan, all 3 patients had progression of disease (Figure 7). The first patient, a 27-year-old male, received irinotecan as a single agent (250 mg/m^2^) every 3 weeks. He had stable disease as a best response to therapy, with a slight interval decrease of several lesions, including a large lung metastasis at week 6, but developed clinical progression of a spinal metastasis after month 3 and was taken off this therapy to pursue spinal radiation. The second patient, a 48-year-old male, received infusional 5-fluorouracil plus irinotecan (180 mg/m^2^) every 2 weeks. He achieved stable disease at month 3 but developed clinical progression shortly afterward, with new large-volume ascites, and subsequently discontinued cancer therapy. The third patient was a 19-year-old woman, who received irinotecan (200 mg/m^2^) every 3 weeks with bevacizumab (10 mg/kg) every 3 weeks, as well as gemcitabine (1000 mg/m^2^) on days 1 and 8, every 3 weeks. This patient had an unconfirmed partial response at month 3 (–30% by Response Evaluation Criteria in Solid Tumours [RECIST 1.1]). However, irinotecan was discontinued after 4 months due to gastrointestinal toxicities, and her lesions progressed in the following months. These clinical reports provide preliminary clinical evidence that irinotecan has clinical activity in advanced FLC. However, rational systemic combinations will be necessary to improve the breadth and durability of responses in advanced FLC. The findings reported in this study lay a strong foundation to rationally combine DT2216 and irinotecan for a more effective treatment of FLC.

## Discussion

Combination therapeutics offer many advantages in cancer therapy, enhancing efficacy over single agents and reducing the emergence of drug resistance. In particular, combination therapies are advantageous in addressing the genetic, phenotypic, and therapeutic heterogeneity found in cancer. However, synergistic anticancer combinations can also be synergistically toxic to the patient, so considerations of overlapping side effects must also be prudently weighed. We probed for efficacious combinations from a recent drug-repurposing screen and identified dual inhibition of TOPO1 with irinotecan and BCL-XL with DT2216 as a promising and clinically viable approach to treat FLC, a rare tumor with no proven effective systemic therapies.

One of the top hits of a recent drug-repurposing screen comparing FLC models against PHH was SN38 (14). There, we observed continued consistent enhanced sensitivity of FLC cells dissociated from PDX and directly from patient tumors to SN38. Based on these preliminary findings (14), some clinicians have begun to administer irinotecan as a salvage option for patients with advanced FLC. We reviewed a patient- and community-directed medical registry, the Fibrolamellar Registry, and identified 3 cases in which irinotecan was administered, and all cases clinical benefit was reported.

We hypothesize that FLC-specific tumoral downregulation of uridine diphosphate glucuronosyl transferase 1A1 (UGT1A1) may result in a unique tumoral susceptibility to this agent. Irinotecan, a TOPO1 inhibitor widely used in pediatric and adult cancers, is converted in the liver by carboxylesterases to SN38, which is 100–1,000 times more potent than its prodrug form. SN38 is then deactivated in the liver by UGT1A1 into an inactive metabolite, SN38 glucuronide (29). A possible reason for the sensitivity for SN38 in FLC over normal liver cells or PHH is the downregulation of the UGT1A1 in FLC. UGT1A1 is decreased at the transcriptome level in FLC (log_2_= –1.68) (11). This increases the accumulation of SN38 and particularly extends the drug lifetime in tumor cells. Consistent with this mechanism, patients with Gilbert’s syndrome, who are lacking members of the UTG1A family, are sensitive to irinotecan (30, 31). Notably, many of the top hits from the drug repurposing screen were HDAC inhibitors. These HDAC inhibitors also undergo metabolism via the UGT1A family, and this could contribute to their selectivity for FLC over PHH.

Another top hit from the drug repurposing screen was navitoclax, an inhibitor of the antiapoptotic proteins Bcl-2 and BCL-XL. While navitoclax synergized strongly with SN38 in our prior study, we felt that DT2216 was a more promising BCL-XL inhibitor, given its greater specificity for BCL-XL over Bcl-2, along with an attenuated effect on platelet counts. Notably, our in vivo safety testing confirmed that thrombocytopenia caused by clinically relevant doses of DT2216 (42%–61% platelet decrease) was substantially less than that caused by navitoclax (97% decrease). An anchor screen determined that DT2216 synergized with HDAC inhibitors fimepinostat and panobinostat, as well as the TOPO1 inhibitor SN38.

We decided to advance preclinical combination studies of DT2216 with irinotecan based on the aforementioned unique susceptibility of irinotecan to FLC, both mechanistically and in the limited case series, as well as the broad potential application of this combination in both adult and pediatric cancers where irinotecan is an established active agent. In vitro screening of the combination of DT2216 and SN38 revealed an additive or synergistic effect in cells dissociated from 4 independently derived FLC PDX lines. Four validated FLC PDX models were leveraged to explore different dosing strategies, and we concluded that a lower-dose protracted regimen of irinotecan had the most promising safety and efficacy profile. All FLC PDX demonstrated consistent therapeutic benefit to irinotecan, which was augmented when combined with DT2216. In the PDX 32 line, which is most representative of a larger collection of FLC PDX, there was complete response that was durable even after discontinuation of therapy. We anticipate that tumoral levels of UGT1A1 and BCL-XL may be predictive biomarkers of response to this particular combination.

In conclusion, despite broad resistance to a curated panel of currently clinically utilized chemotherapies and targeted agents, FLC appears to be sensitive to TOPO1 inhibition, particularly with irinotecan and its active metabolite SN38. We identified upregulation of the prosurvival Bcl-2 family protein, BCL-XL, in resistant FLC models and demonstrated that modulating apoptotic priming with the BCL-XL targeting agent navitoclax resulted in synergy with irinotecan, particularly in resistant FLC models (14). Here, these findings were recapitulated with the BCL-XL–targeting PROTAC DT2216, which offered an improved safety profile in comparison with navitoclax. Indeed, combination with irinotecan and DT2216 at clinically achievable dosing provided durable responses in vivo even after withdrawal of therapy. Finally, we reviewed a patient- and community-based FLC registry and uncovered 3 patients with advanced FLC treated with irinotecan off label; all had some degree of clinical benefit. Based on this work, a clinical trial of DT2216 and irinotecan is currently in development.

## Methods

### Human tissue samples.

Areas of the resection — both tumor tissues or adjacent nontumor liver tissue, if available — that were not needed for diagnosis and treatment were collected for the study. Tissue collected for the study were placed in cold sterile PBS on ice and cut into 2–3 cm × 0.5 cm portions and placed in 50 mL tubes of Roswell Park Memorial Institute medium (Thermo Fisher Scientific, RPMI 1640 + glutamine) supplemented with 2% penicillin/streptomycin (Thermo Fisher Scientific). The tumor tissue was prepared by removing any connective tissue, blood vessels, blood clots, and necrotic tissue, which was then discarded. Pieces of tissue were divided to be either fixed in 10% formalin (Thermo Fisher Scientific) for histologic analysis, flash frozen in liquid nitrogen or dry ice, frozen in OCT compound (Thermo Fisher Scientific), or frozen in RNALater solution (Qiagen) for protein and RNA analysis. Some pieces were cut into 2 mm pieces for implantation into mice. The rest of the tumor pieces not utilized for sample analysis or mice implantation were cut into 2 mm pieces and prepared for tumor dissociation. Dissociated human tumor cells were used for direct-from-patient screening or implantation into mice. The diagnosis of FLC was determined by experienced pathologists via histologic analysis, demonstration of the *DNAJB1-PRKACA* fusion transcript via reverse transcriptase PCR (RT-PCR), and DNAJB1-PRKACA fusion protein via Western blot.

### Tumor dissociation.

Tumor tissue was cut into 2 mm pieces in RPMI on ice with the connective tissue, blood vessels and blood clots, and necrotic tissue discarded. These pieces were placed into 50 mL Falcon tubes of RPMI (+2% penicillin/streptomycin), collagenase V (Worthington; 1 mg/mL), neutral protease (Worthington; 0.5 U/mL), and DNase (Roche; 1 μg/mL). The tissue was digested while rotating at 37°C (Benchmark Scientific Roto-Therm) until digestion was complete. Digested tissue was strained through a 200 μm strainer (Pluriselect) using a syringe plunger on the remaining undigested pieces. The digested tissue was then pushed through a 100 μm strainer (Thermo Fisher Scientific), and the cells were spun down at 300*g* for 5 minutes at 4°C. The supernatant was removed, and the pellet was depleted of RBCs via 10-second resuspension in 1 mL of water followed by 49 mL of 1× PBS. The cells were then spun down at 300*g* for 5 minutes at 4°C, resuspended in RPMI (+2% penicillin/streptomycin), and counted using a hemocytometer. The cells were then used for either a drug response screening, implantation into mice, or for in vitro experiments. Cells derived from PDX and used for in vitro experiments were subjected to mouse cell depletion according to the manufacturer’s instructions (Miltenyi Biotec).

### Mice.

NSG mice (NOD.Cg-Prkdcscid Il2rgtm1Wjl/SzJ, strain 005557), purchased from The Jackson Laboratory and bred at the Rockefeller University animal facility specific pathogen–free (SPF) immune core, were used for the in vivo experiments. Mice were kept in a 12-hour light/dark cycle and had ad libitum access to food and water. NSG mice were fed a diet of Modified PicoLab Mouse Diet w/0.12% amoxicillin. Both male and female 4- to 8-week-old mice were used for implantation of tumors and passaging of PDX. Female NSG mice 5–8 weeks old were used for in vivo drug studies. PDX mice were monitored for their tumor growth and health twice a week. Mice that had tumors approximately 2 cm wide or displayed signs of illness were sacrificed, with the tumor removed and passaged into naive NSG mice.

### Mice passaging and implantation.

Mice showing tumor growth or signs of illness were sacrificed using a lethal dose of ketamine/xylazine and cervical dislocation. Pieces of the tumor were cut and placed in RPMI on ice. Mice used for passaging were anesthetized using isoflurane and given buprenorphine for analgesia. The pieces were implanted either s.c., under the kidney capsule, or directly into the liver. A small incision in the skin of each mouse was made in the flank area, and pieces implanted s.c. were placed between the skin and fascia; the skin was closed using staples. For implantation directly into the liver, the left liver lobe was exposed via an abdominal incision just below the xiphoid process. A small area on the surface of the liver was cauterized, and using thin forceps, a small tunnel in the liver parenchyma was made in the area. A narrow piece of tissue was placed into the tunnel, and the tunnel opening was then cauterized; the liver was replaced in the abdominal cavity. The fascia was sutured, and the skin was stapled together. For implantation under the kidney capsule, the kidney was exposed via a paraspinal approach. A small incision of the kidney capsule was made, and a small piece of tissue (<0.5 mm) was placed under the kidney capsule and advanced from the incision. The kidney was repositioned, the fascia was sutured, and the skin closed with staples.

Cells dissociated from PDX or patient tumors were injected s.c., into the liver, or into the spleen. For cells injected s.c., about 5 × 10^5^ to 2 × 10^6^ cells with RPMI were mixed at a 1:1 ratio with Matrigel (Corning) and injected s.c. in the flank or abdominal area. About 5 × 10^5^ cells mixed at a ratio of 1:1 with Matrigel were injected directly into the left lobe of the liver for intrahepatic injections. For splenic injections, about 5 × 10^4^ to 5 × 10^5^ cells in RPMI were injected directly into the spleen.

### Drug formulation for in vivo treatments.

Irinotecan (Selleckchem, S2217) and navitoclax (ChemieTek, CT-A263) were diluted in DMSO to make stock solutions, were aliquoted, and were stored at –80°C. DT2216, both the i.p. (nonclinical) and i.v. (clinical) formulations, were formulated as previously reported (20). The clinical formulation of DT2216 was diluted 1:1 in 5% dextrose to achieve a higher volume of injection in mice. Irinotecan was formulated in 99.5% of 0.9% sodium chloride (Thermo Fisher Scientific) and vortexed. Navitoclax was formulated in 10% ethanol (Thermo Fisher Scientific), 30% PEG-400 (MilliporeSigma), and 60% Phosal 50PG (Lipoid), with vortexing after adding each component. Working solutions of irinotecan and navitoclax were made fresh prior to each administration.

### In vivo drug testing.

Female NSG mice aged 5–8 weeks were used for in vivo studies. Approximately 5 × 10^5^ to 1 × 10^6^ FLC tumor cells from dissociation were injected s.c. in the abdominal region. Mice were then followed biweekly, and treatment was initiated when tumors reached an average volume of 150 mm^3^ via measurement by caliper. Navitoclax was given at 50 mg/kg daily p.o. for 14 consecutive days, with a break on the third week, for a total of 21 days as a single treatment cycle. Irinotecan was given at 2.5–5 mg/kg daily by i.p. injections, 5 days on and 2 days off, and a break on the third week, for a total of 21 days for a single treatment cycle. The nonclinical formulation of DT2216 was given at 15 mg/kg by i.p. injections once or twice a week for 3 weeks as a single treatment cycle. The clinical formulation of DT2216 was given at 15 mg/kg by i.v. injection via the tail vein once or twice a week for 3 weeks as a single treatment cycle.

Mice received supportive care with s.c. injection of saline and oral Nutra-Gel AIN-93 diet bacon flavor (Bio-Serv) or MediGel Hazelnut (ClearH_2_O) throughout the treatment. Mice were monitored daily for their health and weight. Tumor size was measured initially at day 0 by electronic calipers and then twice a week every Monday and Thursday until the end of treatment or during the monitoring phase after treatment. Tumor volumes measured by electronic caliper were calculated as (length × width^2^)/2. Tumors were extracted from the mice and photographed. The tumors were then divided into pieces to be fixed in 10% formalin, frozen as OCT blocks, or flash frozen for further analysis.

### In vivo platelet toxicity assay.

Twenty female 5- to 8-week-old NSG mice with FLC tumors injected s.c. were used in this study and treated starting at day 1 to end of treatment on day 4. There were 4 different groups in the study, with 5 mice in each group. The vehicle group received vehicles for both DT2216 (i.v.) and navitoclax (p.o.). The mice in the DT2216 nonclinical formulation group were each treated with a single i.p. dose of the nonclinical formulation of DT2216 (15 mg/kg). The mice in the DT2216 clinical formulation group were each given a single i.v. dose of the clinical formulation of DT2216 (15 mg/kg) via tail vein. The mice in the final navitoclax group received a daily p.o. treatment of navitoclax (50 mg/kg). All treatments started at day 1. Approximately 100 μL of blood was collected from each mouse on day 1 prior to treatment via submandibular plexus route in EDTA tubes (BD Biosciences) and collected again within 6 hours after last navitoclax treatment on day 4. Platelets were counted using a veterinary hematology analyzer Element HT5 (Heska).

### In vivo DT2216 and irinotecan combination therapy in naïve mice.

Twelve naive female NSG mice aged 5–8 weeks with no tumors implanted were used for in vivo studies to assay the safety of the DT2216 and irinotecan therapy for 1 treatment cycle. There were 3 different treatment groups with 4 mice in each group in the study: vehicle group, irinotecan only group, and combination treatment group. The vehicle group received vehicles for both DT2216 (i.v.) and irinotecan (i.p.). Irinotecan was given at 5 mg/kg daily via i.p. injections for 5 days on and 2 days off, with a break on the third week, for a total of 21 days in a single treatment cycle. The clinical formulation of DT2216 was given at 15 mg/kg by i.v. injection via the tail vein twice a week for 3 weeks as a single treatment cycle. After receiving treatment for 14 days, 2 mice from each group were sacrificed, approximately 100 μL of blood was collected in EDTA tubes (BD Biosciences), and 500 μL of blood was collected in capillary blood collector tubes (BD Biosciences) for serum; blood was subjected to a complete blood count (CBC) analysis and liver panel analysis, performed at Memorial Sloan Kettering Cancer Center in the Laboratory of Comparative Pathology department.

### In vivo BCL-XL degradation assay.

NSG PDX mice (5–8 weeks old) were given a single dose of either the i.v. formulation or the i.p. formulation of DT2216 (15 mg/kg), were given the vehicle, or were given no treatment. Tumors and liver samples were harvested from mice, with each single mouse representing an individual time point on days 2, 4, 8, and 12. Pieces of the tumor and liver were frozen for each time point and lysed to measure the BCL-XL degradation levels. A Western blot was performed after extracting the protein from the samples, and a densiometric analysis was performed (LI-COR).

### Drug screening and cell viability assay.

Cells dissociated from FLC PDX tumors were depleted of mouse stromal cells and used for high-throughput screening. Cells were plated into either 384-well plates (Grenier Bio One) or a 96-well plate containing screening compounds at 2,000 cells per well (384-well format) or 20,000 cells per well (96-well format) in Kubota’s medium (Phoenix Songs Biologicals) supplemented with 2% penicillin/streptomycin. Each plate contained negative control wells with DMSO (I_Negative_) and positive control wells (I_Positive_) treated with 20 μM chaetocin (Selleckchem, S8068). Cells plated with the compounds (I_Compound_) were incubated for 48–72 hours at 37°C. After incubation, CellTiter-Glo reagent (Promega) was added at a 1:1 ratio to the total volume with cells and compounds. The plates were read for luminescence (BioTek Synergy Neo). The normalized percent cell survival was calculated as:
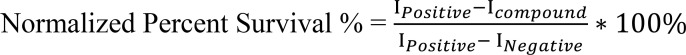


Dose-response curves were generated with GraphPad PRISM (version 9). Similarly, the compounds were screened against PHH) grown in humanized mice as described previously (14). PHH cells were plated at 50,000 cells per well in W10 media (William’s E media supplemented with ITS [BD Biosciences], penicillin/streptomycin/ciprofloxacin). PHH cells were incubated and assayed for viability as described for FLC PDX.

### Synergy assay.

FLC PDX tumors were dissociated into cells, depleted of mouse stromal cells, and used for synergy assays. Compounds were plated in a 2-compound combination matrix on either a 384-well plate or 96-well plate. Compound A (DT2216) and Compound B were plated with concentrations ranging from 10 nM to 10 μM. Each plate contained negative control wells with DMSO only, as well as positive control wells with 20 μM chaetocin. Each plate also contained control wells treated with single-agent compounds. Depleted PDX cells were plated at 2,000 cells/well (384-well format) or 20,000 cells/well (96-well format) in Kubota’s StemCell Growth Medium (Phoenix Songs Biologicals) with 2% penicillin/streptomycin. Cells were incubated with compounds for 72 hours and assayed for luminescence with the CellTiter-Glo reagent (Promega) according to the manufacturer’s instruction. PHH cells were incubated and assayed for viability as described for FLC PDX. The results were assessed using SynergyFinder 2.0 (27). Each PDX line was evaluated against a range of selected agents, and the ZIP scores from synergistic areas of the matrix of drugs were quantified. Efficacies were compared at 1 μM and at a 1:1 ratio for DT2216/drug.

### PDX models express DNAJB1-PRKACA.

For the following in vitro and in vivo testing, we used PDX that we had previously developed as models for FLC. Fresh tumor tissue was processed into pieces and implanted s.c. or dissociated into single cells and injected s.c. in NSG mice, without passage in vitro. One PDX mouse lines was derived from a primary liver tumor (PDX 31), and 3 were derived from metastases (PDX 32, PDX 33, and PDX 34).

Each PDX line was validated based on 4 criteria (14). First, the PDX were tested for the presence of the *DNAJB1-PRKACA* fusion transcript, a hallmark of the majority of FLC cases. RNA was extracted and the presence of the fusion transcript was verified using RT-PCR (Supplemental Figure 1A). Both the WT *PRKACA* and *DNAJB1-PRKACA* transcripts were clearly detected in lysed tumor samples. Second, using Western blotting, extracts were tested for the presence of both PRKACA and DNAJB1-PRKACA, using an antibody that recognizes the common carboxyl terminus of both proteins (Supplemental Figure 1B). As shown previously, the ratio of DNAJB1-PRKACA/PRKACA is variable among different patients (Supplemental Figure 1C) (5). Third, the histopathology of each PDX was compared with the original patient tumor to verify similarity (14). Fourth, it was determined whether the transcriptome of the PDX recapitulated the transcriptome of its tumor of origin (14).

### Immunoblotting.

Tissue samples from PDX were suspended in lysis buffer A (50 mM Tris pH 7.5, 150 mM NaCl, 0.5% Na deoxycholate, 1% Triton-X, 1 mM EDTA, 5 mM EGTA, cOmplete Mini EDTA-free Protease Inhibitor Cocktail [Roche], PhosSTOP phosphatase inhibitor cocktail [Roche]). Samples were then sonicated on ice and centrifuged at 4°C for 15–30 minutes at 30,130*g*. The supernatant was collected, and we measured the protein concentration by a modified Lowry assay (DC protein assay, Bio-Rad). Samples were diluted in a 4× Nupage LDX sample buffer, heated at 100°C for 5 minutes, and then loaded on 4%–12% Bis-Tris gels (NuPage, Invitrogen); they were run in MOPS buffer for 50 minutes at 200 V, as per manufacturer recommendations.

For immunoblotting, we transferred the gels to nitrocellulose membranes using iBlot (Invitrogen). We blocked the membrane in 5% milk in TBST. Membranes were incubated with the primary antibody (at the recommended dilution; Cell Signaling Technology) overnight, with rocking at 4°C, and were probed the next day with the appropriate secondary antibody and Amersham ECL Prime HRP detection kit (GE Healthcare). Membranes were developed on the LI-COR developer.

The antibodies used included anti–GAPDH mouse monoclonal antibody (catalog GTX627408) from GeneTex; anti–BCL-XL (54H6) rabbit mAb (catalog 2764S) from Cell Signaling Technology; and anti–PRKACA (Pka C α) (D38C6) rabbit mAb (catalog 5842S) from Cell Signaling Technology.

### Calculation of TGI rate.

Mean TGI rate was used to quantify the drug response of the different treatment arms compared with the control group at different time points. V_T,t_ and V_T,0_ represent the mean tumor volume at the time t and time 0 of the treatment arm, while V_C,t_ and V_C,0_ represent the mean tumor volume of the vehicle control arm (32, 33).
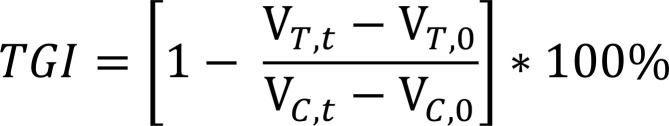


### Response categorization.

Drug response was calculated to label the drug response of each treatment group. Tumor volumes were compared at time (V_t_) with the initial measurement (V_0_), 
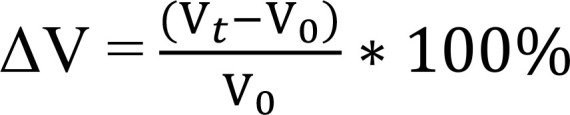


Within each group, we identified the minimum ΔV = V_min_ and the mean average ΔV as V_mean_. The criteria for response (mRECIST), adapted from RECIST criteria (34, 35), were defined as in Table 1.

### Statistics.

The statistical analysis and graphs were prepared using GraphPad Prism 9 (GraphPad Software), SAS Studio 3.8, and Microsoft Excel. For analyzing the means of 3 or more groups, we used 1-way ANOVA tests. For comparing the means of 2 groups, we used a 2-sided unpaired *t* test. A linear mixed-effects regression model was used to compare the growth trajectories between the different treatment arms and the control over the treatment and monitoring phases. The model included group, day (categorical), and group × day interaction as fixed effects and mouse as a random effect. A significant group × day interaction indicated a difference in tumor growth over time between groups. Tumor volume was log transformed prior to analysis. *P <* 0.05 was considered statistically significant.

### Study approval.

With the approval of the Rockefeller University IRB (no. SSI-0797), written informed consent was obtained from each patient prior to clinically indicated tumor resections at collaborating institutions. All studies were conducted in accordance with recognized ethical guidelines. All mouse work was done with the approval of the Rockefeller University IACUC (no. 20027-H).

## Author contributions

Conceptualization was contributed by BS, GL, MVO, and SMS. Methodology was contributed by BS, GL, MVO, RDM, YDJ, CMR and SMS. Investigation was contributed by BS, DN, GL, TMF, RDM, and MY. Visualization was contributed by BS, DN, and SMS. Synthesis and characterization of DT2216 was done by GZ, PZ, and DH. Curation and analysis of patient data was done by RDM and SMS. Statistical analysis was done by CSJ, AQ and RV. Funding acquisition was contributed by SMS. Project administration was contributed by SMS. Supervision was contributed by SMS. Writing of the original draft was contributed by BS, DN, and SMS. Review and editing of the manuscript was contributed by BS, CMR, DN, GL, CR, CSJ, AQ, MY, YPDJ, PC, MVO, DZ, and SMS. There are 3 first authors. The work started with GL in the lab. When he left, BS took over for the bulk of the project. Throughout all of the project, DN was the primary coordinator of the mice and evaluating the responses.

## Supplementary Material

Supplemental data

## Figures and Tables

**Table 1:**
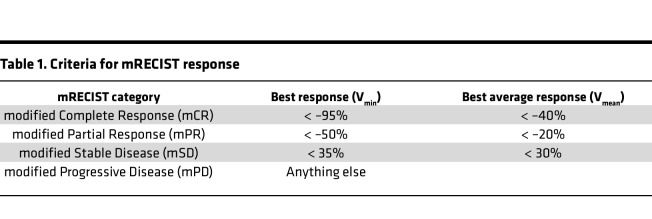
Criteria for mRECIST response

## Version 1 09/08/2022

Electronic publication

## References

[1] Lalazar G, Simon SM (2018). Fibrolamellar carcinoma: recent advances and unresolved questions on the molecular mechanisms. Semin Liver Dis.

[2] O’Neill AF (2021). Fibrolamellar carcinoma: an entity all its own. Curr Probl Cancer.

[3] Graham RP (2015). DNAJB1-PRKACA is specific for fibrolamellar carcinoma. Mod Pathol.

[4] Darcy DG (2015). The genomic landscape of fibrolamellar hepatocellular carcinoma: whole genome sequencing of ten patients. Oncotarget.

[5] Honeyman JN (2014). Detection of a recurrent DNAJB1-PRKACA chimeric transcript in fibrolamellar hepatocellular carcinoma. Science.

[6] Engelholm LH (2017). CRISPR/Cas9 engineering of adult mouse liver demonstrates that the Dnajb1-Prkaca gene fusion is sufficient to induce tumors resembling fibrolamellar hepatocellular carcinoma. Gastroenterology.

[7] Kastenhuber ER (2017). *DNAJB1-PRKACA* fusion kinase interacts with β-catenin and the liver regenerative response to drive fibrolamellar hepatocellular carcinoma. Proc Natl Acad Sci U S A.

[9] Torbenson M (2012). Fibrolamellar carcinoma: 2012 update. Scientifica (Cairo).

[10] Malouf GG (2014). Transcriptional profiling of pure fibrolamellar hepatocellular carcinoma reveals an endocrine signature. Hepatology.

[11] Simon EP (2015). Transcriptomic characterization of fibrolamellar hepatocellular carcinoma. Proc Natl Acad Sci U S A.

[12] Abou-Alfa GK (2020). Phase II multicenter, open-label study of oral ENMD-2076 for the Treatment of Patients with Advanced Fibrolamellar Carcinoma. Oncologist.

[13] El Dika I (2020). A multicenter randomized 3-arm phase II study of (1) everolimus, (2) estrogen deprivation therapy (EDT) with leuprolide + Letrozole, and (3) everolimus + EDT in patients with unresectable fibrolamellar carcinoma. Oncologist.

[14] Lalazar G (2021). Identification of novel therapeutic targets for fibrolamellar carcinoma using patient-derived xenografts and direct-from-patient screening. Cancer Discov.

[15] Ikezawa K (2017). Increased Bcl-xL expression in pancreatic neoplasia promotes carcinogenesis by inhibiting senescence and apoptosis. Cell Mol Gastroenterol Hepatol.

[16] Watanabe J (2004). Prognostic significance of Bcl-xL in human hepatocellular carcinoma. Surgery.

[17] Mason KD (2007). Programmed anuclear cell death delimits platelet life span. Cell.

[18] Vogler M (2011). BCL2/BCL-X(L) inhibition induces apoptosis, disrupts cellular calcium homeostasis, and prevents platelet activation. Blood.

[19] Gandhi L (2011). Phase I study of Navitoclax (ABT-263), a novel Bcl-2 family inhibitor, in patients with small-cell lung cancer and other solid tumors. J Clin Oncol.

[20] Khan S (2019). A selective BCL-X_L_ PROTAC degrader achieves safe and potent antitumor activity. Nat Med.

[21] He Y (2020). DT2216-a Bcl-xL-specific degrader is highly active against Bcl-xL-dependent T cell lymphomas. J Hematol Oncol.

[22] Khan S (2022). BCL-XL PROTAC degrader DT2216 synergizes with sotorasib in preclinical models of KRAS(G12C)-mutated cancers. J Hematol Oncol.

[23] Thummuri D (2022). Overcoming gemcitabine resistance in pancreatic cancer using the BCL-X_L_-specific degrader DT2216. Mol Cancer Ther.

[24] Turnham RE (2019). An acquired scaffolding function of the DNAJ-PKAc fusion contributes to oncogenic signaling in fibrolamellar carcinoma. Elife.

[25] Klaeger S (2017). The target landscape of clinical kinase drugs. Science.

[26] Pettersson M, Crews CM (2019). PROteolysis TArgeting Chimeras (PROTACs) - Past, present and future. Drug Discov Today Technol.

[27] Ianevski A (2020). SynergyFinder 2.0: visual analytics of multi-drug combination synergies. Nucleic Acids Res.

[28] Houghton PJ (1995). Efficacy of topoisomerase I inhibitors, topotecan and irinotecan, administered at low dose levels in protracted schedules to mice bearing xenografts of human tumors. Cancer Chemother Pharmacol.

[29] Oguri T (2004). UGT1A10 is responsible for SN-38 glucuronidation and its expression in human lung cancers. Anticancer Res.

[30] Ando Y (1998). UGT1A1 genotypes and glucuronidation of SN-38, the active metabolite of irinotecan. Ann Oncol.

[31] Iyer L (1998). Genetic predisposition to the metabolism of irinotecan (CPT-11). Role of uridine diphosphate glucuronosyltransferase isoform 1A1 in the glucuronidation of its active metabolite (SN-38) in human liver microsomes. J Clin Invest.

[32] Li Q (2019). DRAP: a toolbox for drug response analysis and visualization tailored for preclinical drug testing on patient-derived xenograft models. J Transl Med.

[33] Zou J (2018). Establishment and genomic characterizations of patient-derived esophageal squamous cell carcinoma xenograft models using biopsies for treatment optimization. J Transl Med.

[34] Therasse P (2000). New guidelines to evaluate the response to treatment in solid tumors. European Organization for Research and Treatment of Cancer, National Cancer Institute of the United States, National Cancer Institute of Canada. J Natl Cancer Inst.

[35] Gao H (2015). High-throughput screening using patient-derived tumor xenografts to predict clinical trial drug response. Nat Med.

