# Supplementary material for: Targeting BCL-XL in fibrolamellar hepatocellular carcinoma
Source: JCI Insight. 2022 Sep 8;7(17):e161820. doi: 10.1172/jci.insight.161820 (PMC9536265; doi:10.1172/jci.insight.161820)
Supplement: Supplemental data [file jciinsight-7-161820-s106.pdf]

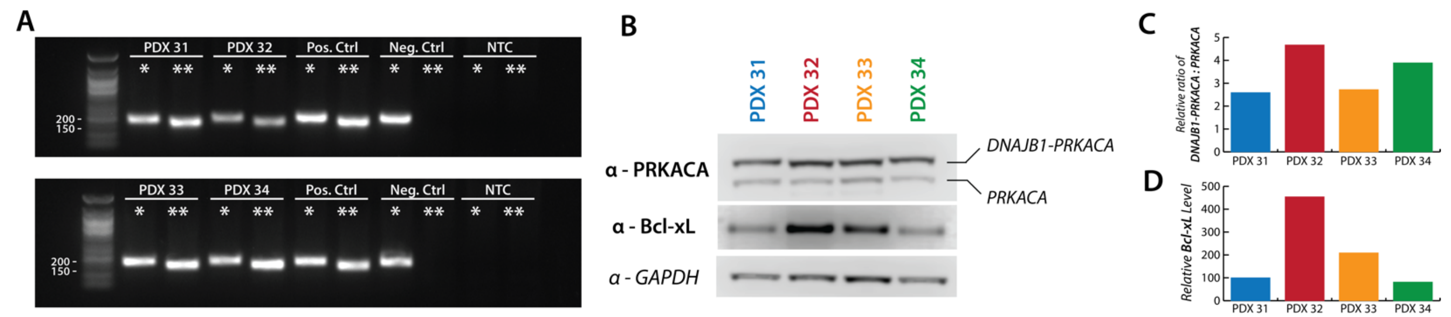

Supplemental Figure 1

Figure S1: A. One-Step RT-PCR of PDX 31, 32, 33, and 34. Wild-type PRKACA (*) amplicon is expected at 184. DNAJB1-PRKACA (**) Amplicon is expected at 160. Pos. Ctrl: Previously validated patient sample. Neg. Ctrl: Hepatocellular carcinoma (HCC) patient sample, contains no DNJB1-PRKACA. NTC: No template control. **(B,C) Fibrolamellar PDXs express DNAJB1-PRKACA and Bcl-xL.** Western blot on tissue from the four PDXs used. α-PRKACA: an antibody that recognized the c-terminus of PRKACA, (also found in the DNAJB1-PRKACA chimera). α-Bcl-xL: an antibody that specifically recognizes Bcl-xL. α-GPADH: Loading control. **C.** The relative ratio of DNAJB1-PRKACA expression to PRKACA in each PDX tumor line. **D.** The level of Bcl-xL relative to GAPDH in each PDX tumor line.

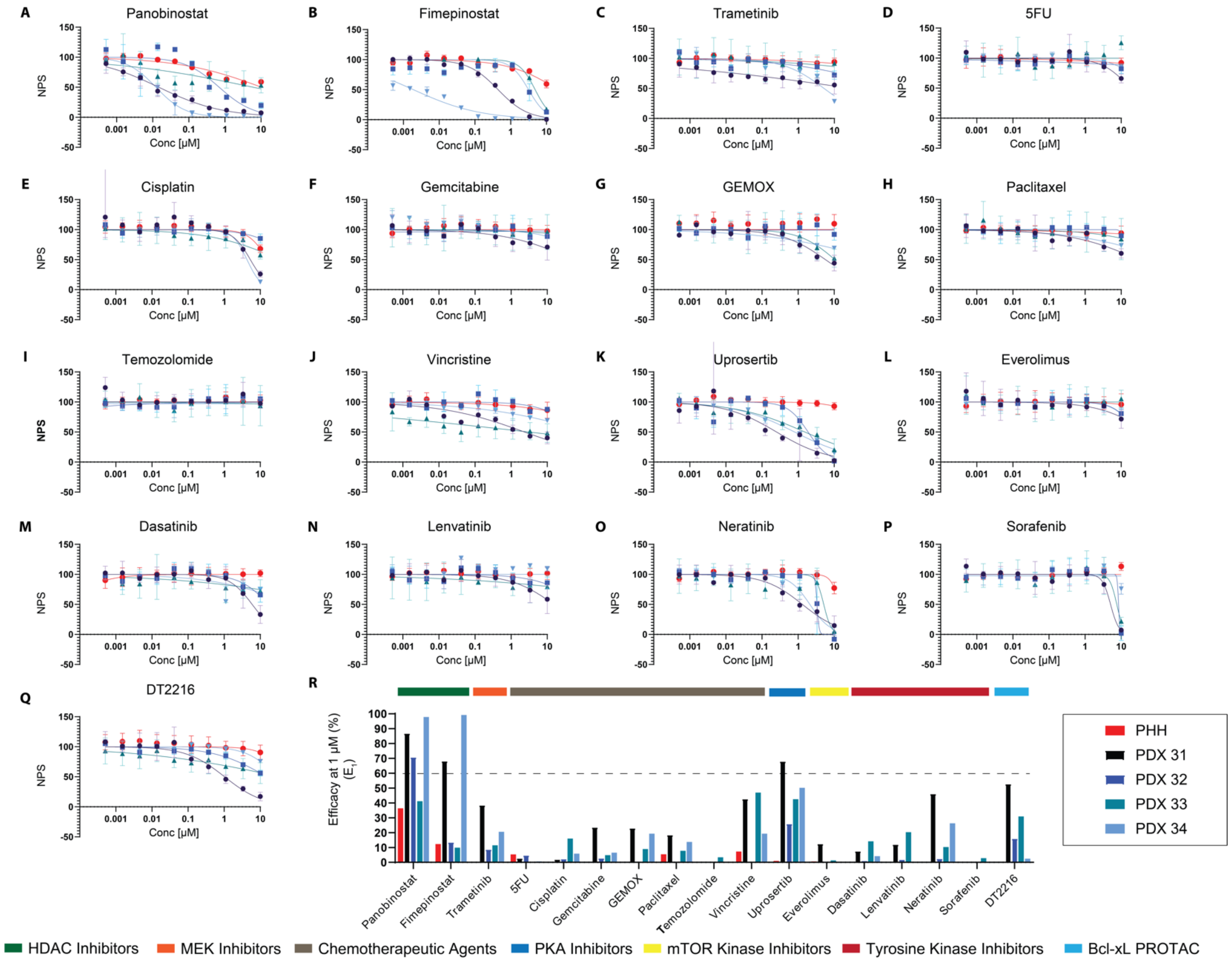

Supplemental Figure 2:

Figure S2: Dose-response curves of selected drugs. A – L: dose-response curves of selected drugs tested against four FLC PDX lines and a PHH control. Drugs were tested at 10 µM - 0.5 nM with 3-fold serial dilution. The y-axis shows normalized percentage survival calculated as [(Positive control – drug response at a given dose) / (Positive control – Negative control)] x 100. The x-axis shows the concentration in µM. **A and B. HDAC inhibitors** (panobinostat and fimepinostat), **C. MEK inhibitors** (trametinib), **D – J. Chemotherapeutic agents** (5-FU: 5-fluorouracil, cisplatin, gemcitabine, GEMOX: gemcitabine and oxaliplatin at a ratio of 10:1, paclitaxel, temozolomide and vincristine), **K. PKA inhibitors** (uprosertib), **L. mTOR kinase inhibitors** (everolimus), **M – P. Tyrosine kinase inhibitors** (dasatinib, lenvatinib, neratinib and sorafenib), **Q. PROTAC** (DT2216: a Bcl-xL specific degrader), Data presented as mean ± SD (n=3) **R.** A plot of the efficacy at 1 µM for all the drugs shown, comparing efficacy in PHH vs FLC PDX tumor lines. **NPS**: Normalized Percentage Survival.

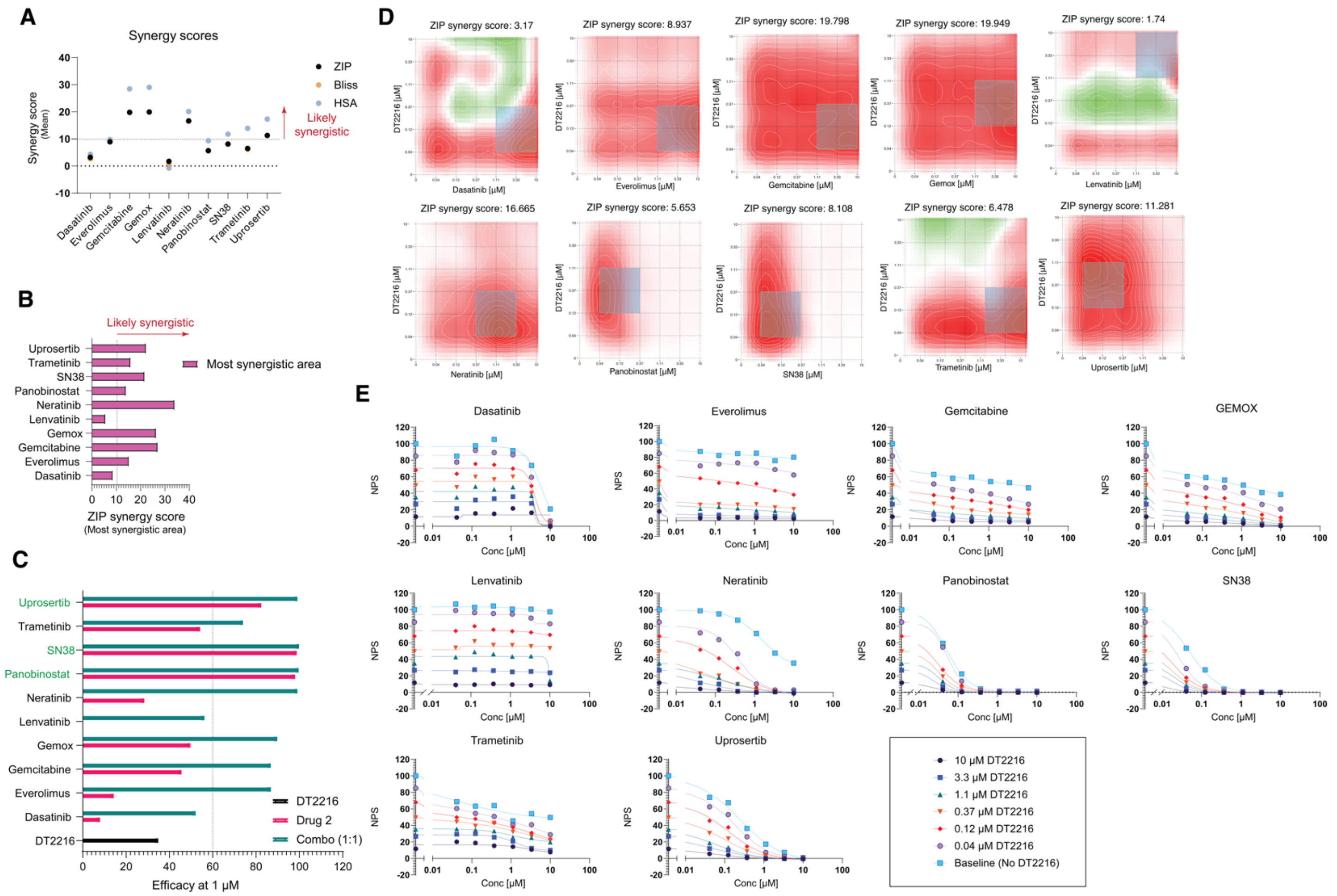

Supplemental Figure 3:

Fig. S3: DT2216 Anchor Screen for PDX 31. A. Synergy scores (ZIP, Bliss and HSA) for DT2216 in PDX 31, **B.** The ZIP scores of the most synergistic area calculated for indicated drugs combined with DT2216, **C.** A plot of the efficacy (100% - Viability) at 1 µM for indicated drugs as single agents or combined with DT2216 (1:1), the dotted line marks the 60% efficacy, **D.** The 6 x 6 dose-response matrices and interaction landscapes of indicated drugs combined with DT2216. Cells were treated for 72 hrs and viability was measured using a cell-titer glo (Promega) assay, **E.** Dose-response curves of therapeutic agents in the screen vs an increasing dose of DT2216. Drugs were tested at 10 µM – 41 nM with 3-fold serial dilution. The y-axis shows normalized percentage survival calculated as [(Positive control – drug response at a given dose) / (Positive control – Negative control)] x 100. The x-axis shows the concentration in µM. The data is an overlay of the baseline dose-response curves from figures 2 and 1S along with the dose-response curves of increasing DT2216 doses. The y-axis is marked with the matching dose-response values for DT2216 in the absence of any other drug.

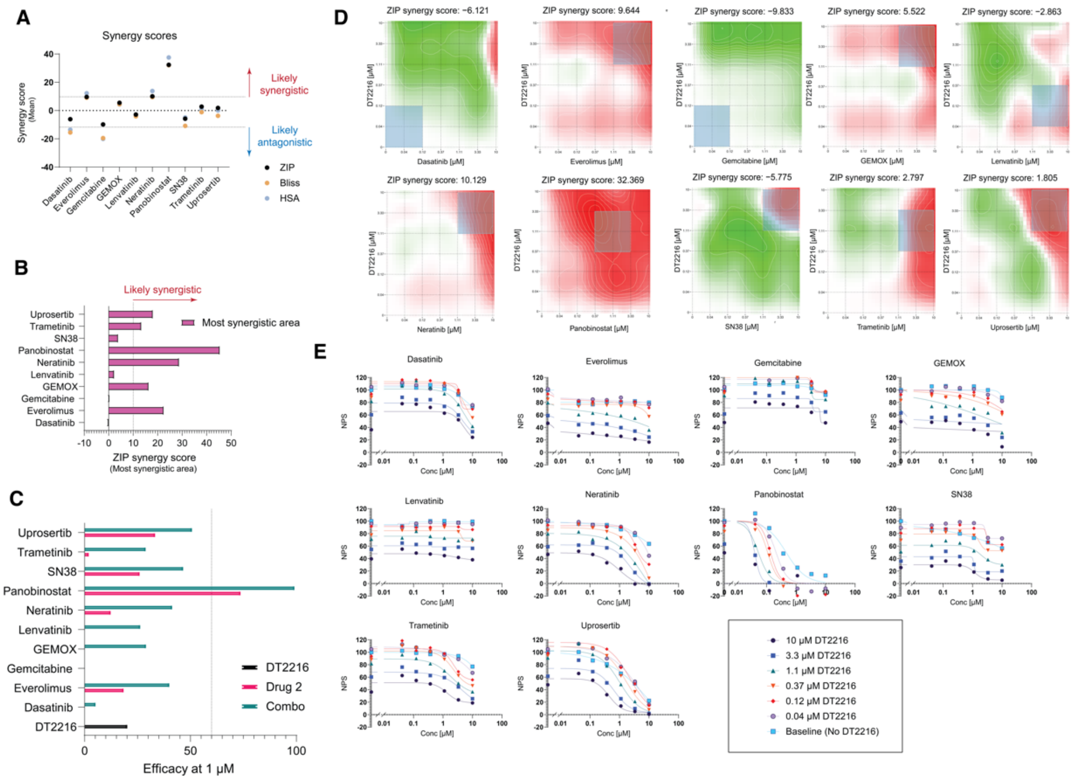

Supplemental Figure 4:

Fig. S4: DT2216 Anchor Screen for PDX 32. A. Synergy scores (ZIP, Bliss and HSA) for DT2216 in PDX 32, **B.** The ZIP scores of the most synergistic area calculated for indicated drugs combined with DT2216, **C.** A plot of the efficacy (100% - Viability) at 1 µM for indicated drugs as single agents or combined with DT2216 (1:1), the dotted line marks the 40% efficacy, **D.** The 6 x 6 dose-response matrices and interaction landscapes of indicated drugs combined with DT2216. Cells were treated for 72 hrs and viability was measured using a cell-titer glo (Promega) assay, **E.** Dose-response curves of therapeutic agents in the screen vs an increasing dose of DT2216. Drugs were tested at 10 µM – 41 nM with 3-fold serial dilution. The y-axis shows normalized percentage survival calculated as [(Positive control – drug response at a given dose) / (Positive control – Negative control)] x 100. The x-axis shows the concentration in µM. The data is an overlay of the baseline dose-response curves from figures 2 and 1S along with the dose-response curves of increasing DT2216 doses. The y-axis is marked with the matching dose-response values for DT2216 in the absence of any other drug.

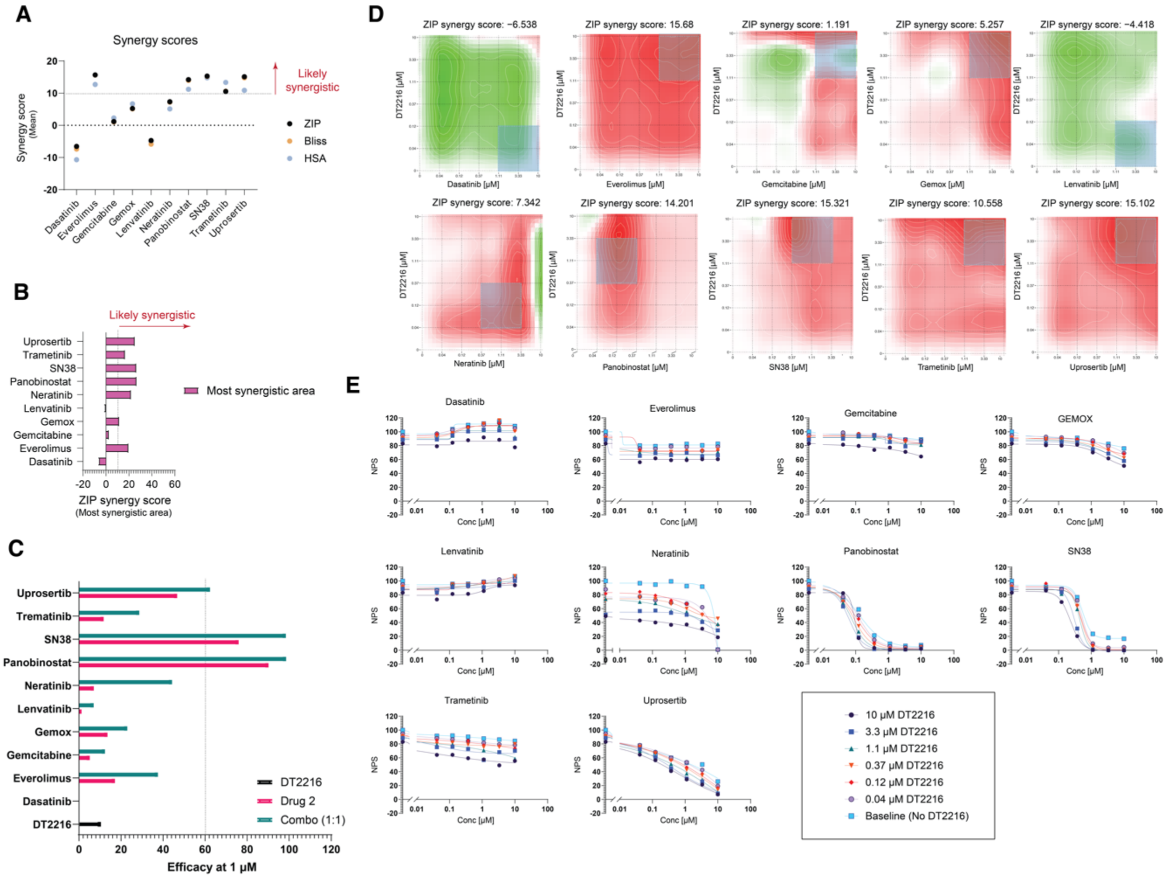

Supplemental Figure 5:

Fig. S5: DT2216 Anchor Screen for PDX 33. A. Synergy scores (ZIP, Bliss and HSA) for DT2216 in PDX 33, **B.** The ZIP scores of the most synergistic area calculated for indicated drugs combined with DT2216, **C.** A plot of the efficacy (100% - Viability) at 1 µM for indicated drugs as single agents or combined with DT2216 (1:1), the dotted line marks the 60% efficacy, **D.** The 6 x 6 dose-response matrices and interaction landscapes of indicated drugs combined with DT2216. Cells were treated for 72 hrs and viability was measured using a cell-titer glo (Promega) assay, **E.** Dose-response curves of therapeutic agents in the screen vs an increasing dose of DT2216. Drugs were tested at 10 µM – 41 nM with 3-fold serial dilution. The y-axis shows normalized percentage survival calculated as [(Positive control – drug response at a given dose) / (Positive control – Negative control)] x 100. The x-axis shows the concentration in µM. The data is an overlay of the baseline dose-response curves from figures 2 and 1S along with the dose-response curves of increasing DT2216 doses. The y-axis is marked with the matching dose-response values for DT2216 in the absence of any other drug.

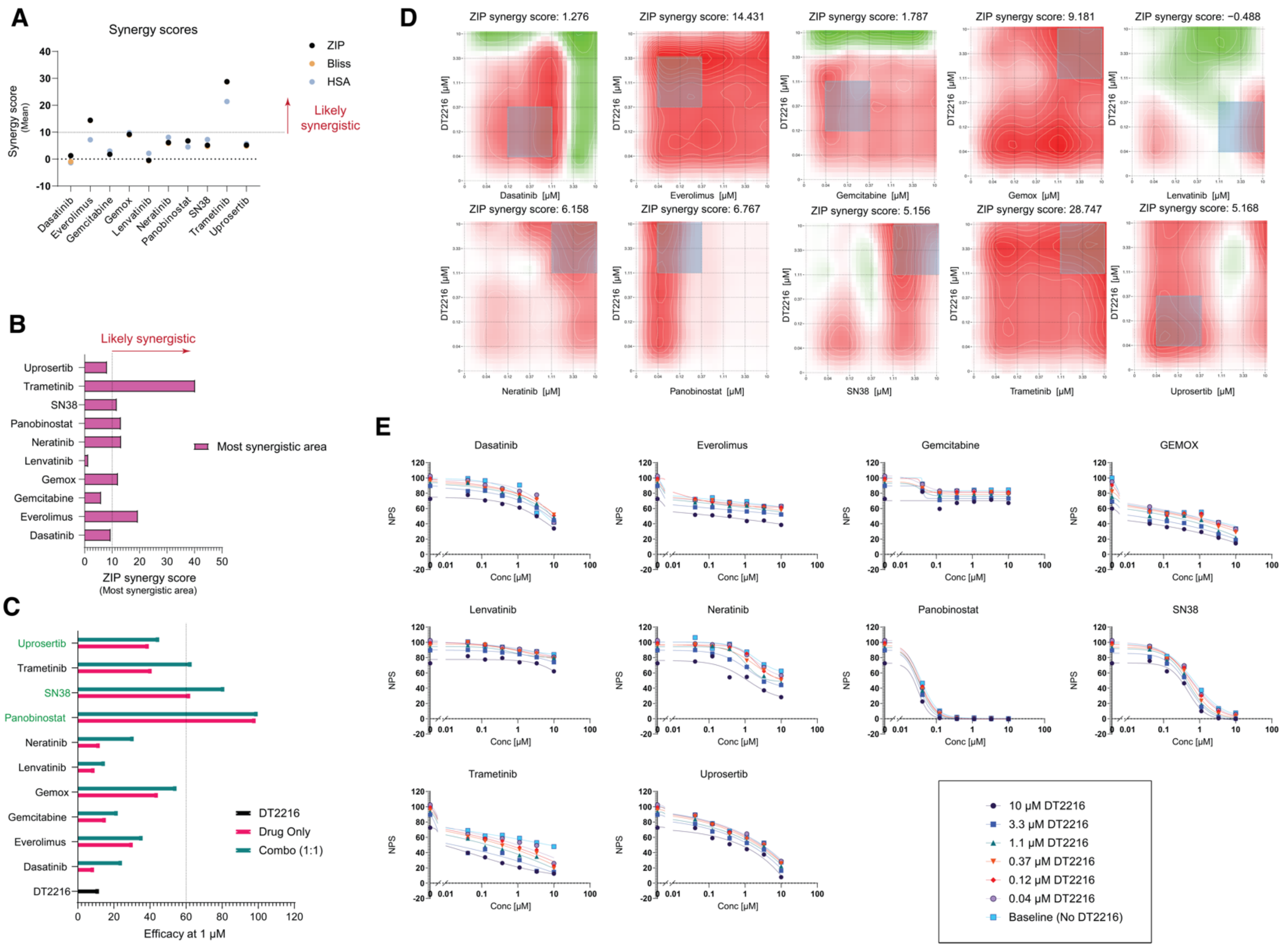

Supplemental Figure 6:

Fig. S6: DT2216 Anchor Screen for PDX 34. A. Synergy scores (ZIP, Bliss and HSA) for DT2216 in PDX 34, **B.** The ZIP scores of the most synergistic area calculated for indicated drugs combined with DT2216, **C.** A plot of the efficacy (100% - Viability) at 1 μM for indicated drugs as single agents or combined with DT2216 (1:1), the dotted line marks the 60% efficacy, **D.** The 6 x 6 dose-response matrices and interaction landscapes of indicated drugs combined with DT2216. Cells were treated for 72 hrs and viability was measured using a cell-titer glo (Promega) assay, **E.** Dose-response curves of therapeutic agents in the screen vs an increasing dose of DT2216. Drugs were tested at 10 μM – 41 nM with 3-fold serial dilution. The y-axis shows normalized percentage survival calculated as [(Positive control – drug response at a given dose) / (Positive control – Negative control)] x 100. The x-axis shows the concentration in μM. The data is an overlay of the baseline dose-response curves from figures 2 and 1S along with the dose-response curves of increasing DT2216 doses. The y-axis is marked with the matching dose-response values for DT2216 in the absence of any other drug.

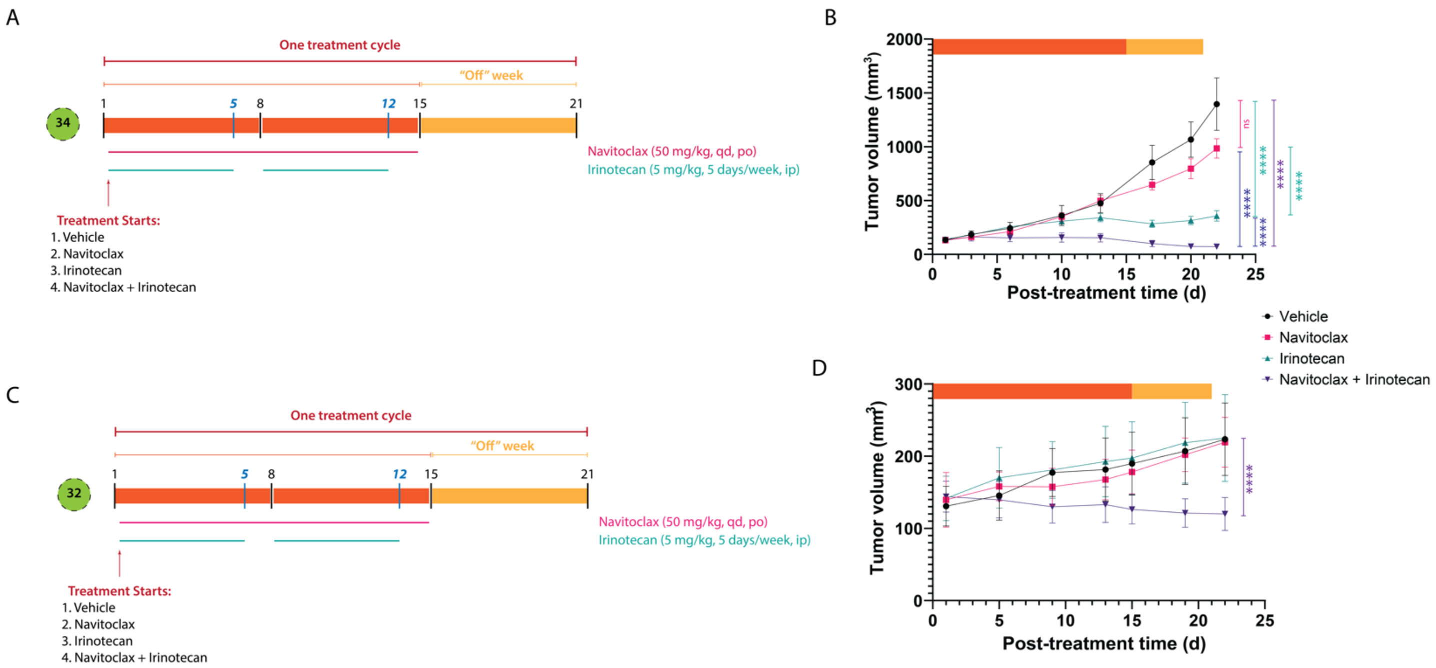

Supplemental Figure 7:

Fig. S7: Navitoclax combined with irinotecan reduces tumor volume in FLC PDXs. A & C. Illustration of treatment timeline for PDX 34 and PDX 32, respectively. Navitoclax was administered daily for two weeks, irinotecan was administered for five days a week for two weeks. The third week the mice are off the treatment. **B & D.** Changes in tumor volume over the course of treatment. Data are presented as the mean ± SEM (n = 3 for vehicle, and n = 4 for other treatment groups at the start of treatment for PDX 34, n = 6 for vehicle and combo treatment groups, n = 5 for other treatment groups at the start of the treatment for PDX 32). ****$p < 0.0001$ in indicated comparisons; **ns**, not significant.

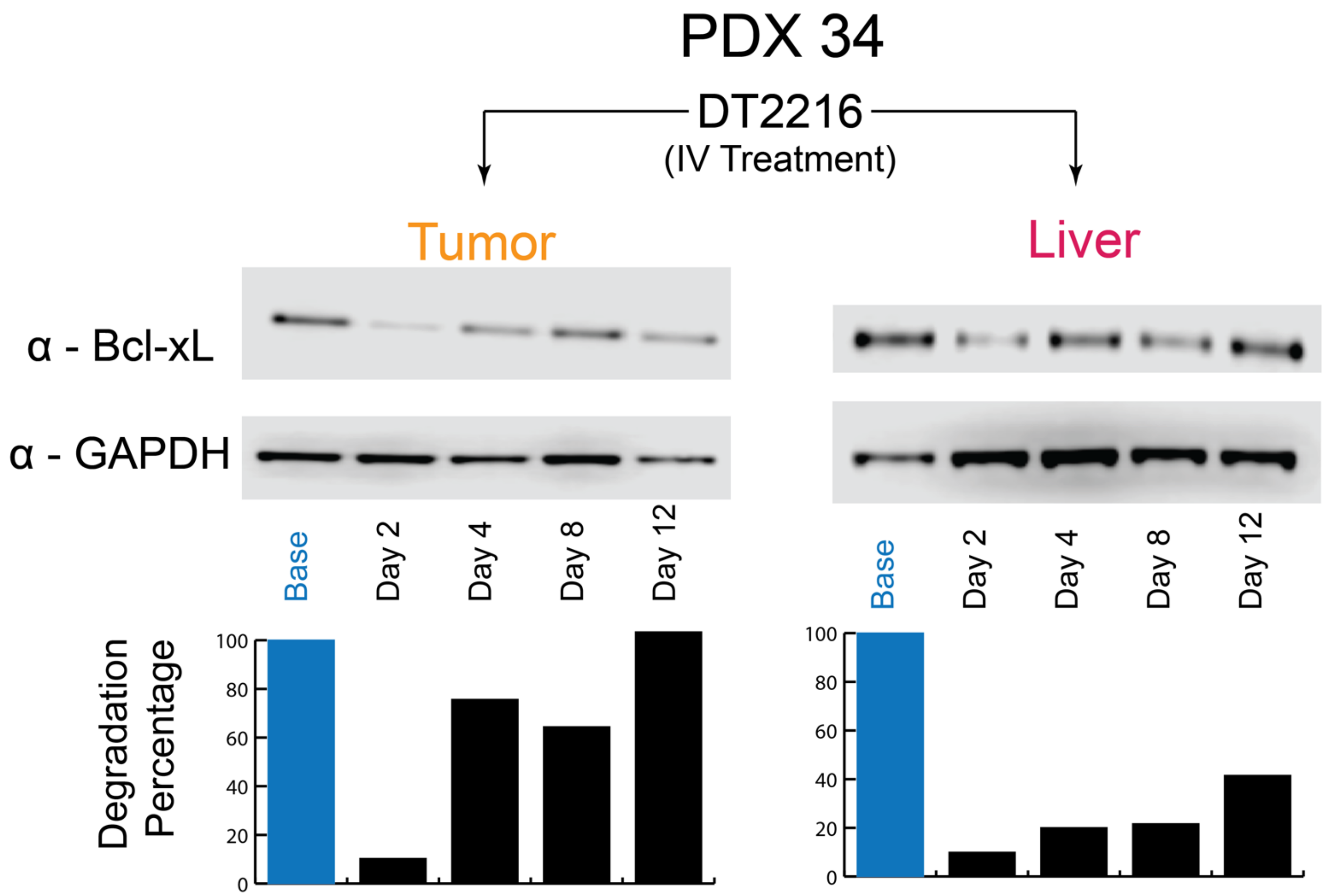

Supplemental Figure 8:

Fig. S8: The IV formulation of DT2216 induces degradation in vivo in FLC PDXs. PDX mice were treated with a single dose of the IV formulation and tumor tissue was collected from the tumor and liver of each mouse. Each time point represents an independent mouse. Bcl-xL level was monitored using Western blotting. GAPDH was used as a loading control for all immunoblot analysis presented. Data were corrected with a normalization factor against GAPDH, and presented as a percentage of the vehicle (Base) treated cells as a control. Upper panel shows the immunoblots, and the lower panel shows the densiometric analysis performed using LI-COR. PDX 34 treated with the IV formulation, tumor and liver samples, respectively.

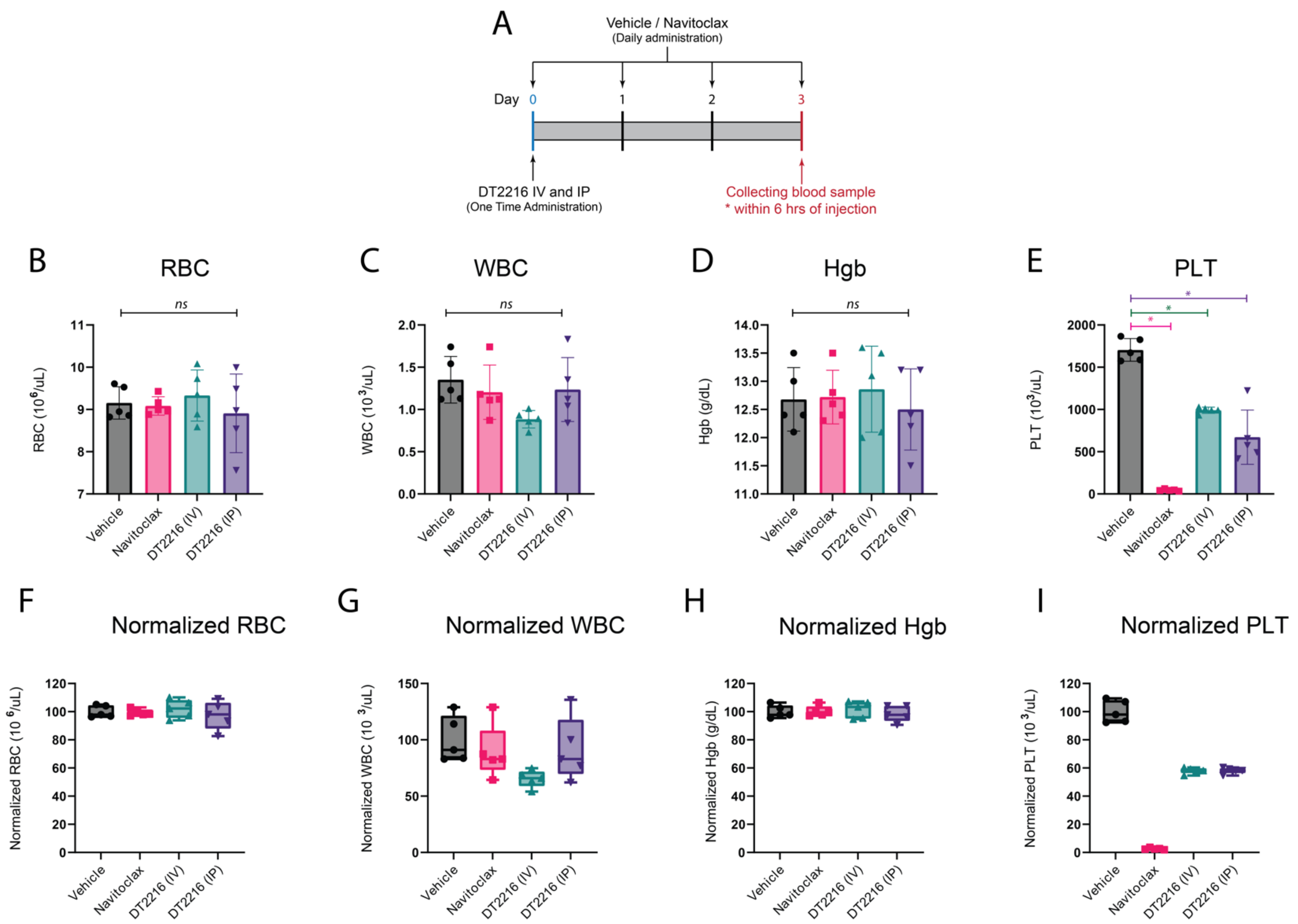

Supplemental Figure 9:

Fig. S9: DT2216 does not cause severe thrombocytopenia. A. Experimental design and timeline of treatment and blood collection, DT2216 was administered at 15 mg per kg body weight, IV: intravenous formulation, IP: intraperitoneal formulation, **B – I.** RBC: red blood cell count, WBC: white blood cell count, Hgb: hemoglobin, PLT: platelet count. **B – E.** Raw count, **F – I.** Count normalized to vehicle control. Data presented as mean ± SD, n = 5 for all groups at the start of the treatment, ***** $p < 0.01$ in indicated comparisons; **ns**, not significant. Each symbol represents data from an individual animal. Statistical significance was determined by one-way ANOVA and two-sided unpaired Student's t-test.

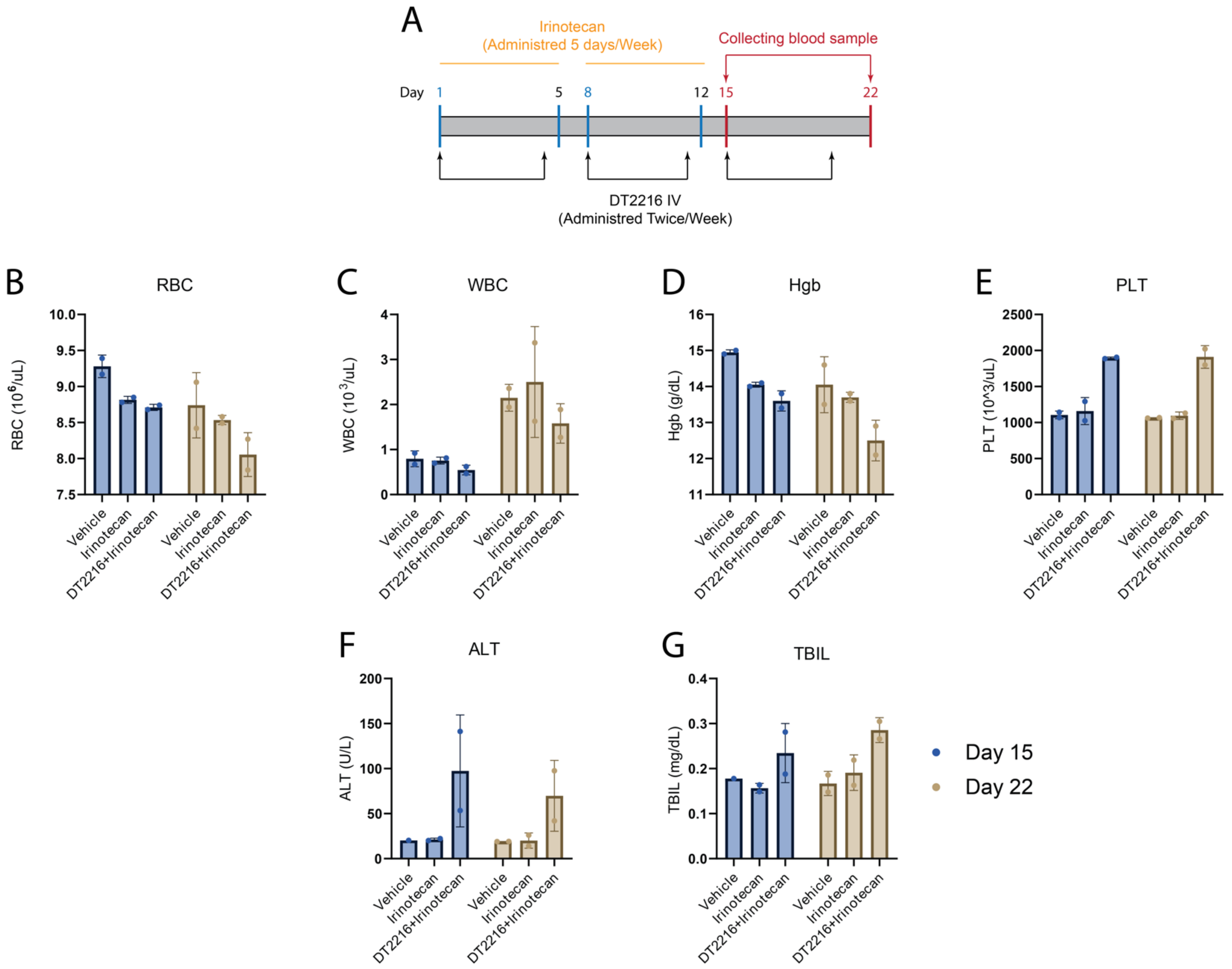

Supplemental Figure 10:

Fig. S10: DT2216 can be safely administered in combination with irinotecan at clinically relevant doses. A. Experimental design and timeline of treatment and blood collection, DT2216 was administered at 15 mg per kg body weight, IV: intravenous formulation, DT2216 was administered twice per week throughout the entire cycle, irinotecan was administered at 5 mg per kg body weight 5 days per week for two consecutive weeks, **B – E.** Raw count of RBC: red blood cell count, WBC: white blood cell count, Hgb: hemoglobin, PLT: platelets count, **F & G.** ALT: Alanine aminotransferase, TBIL: Total bilirubin. Data presented as mean ± SD (n=2)

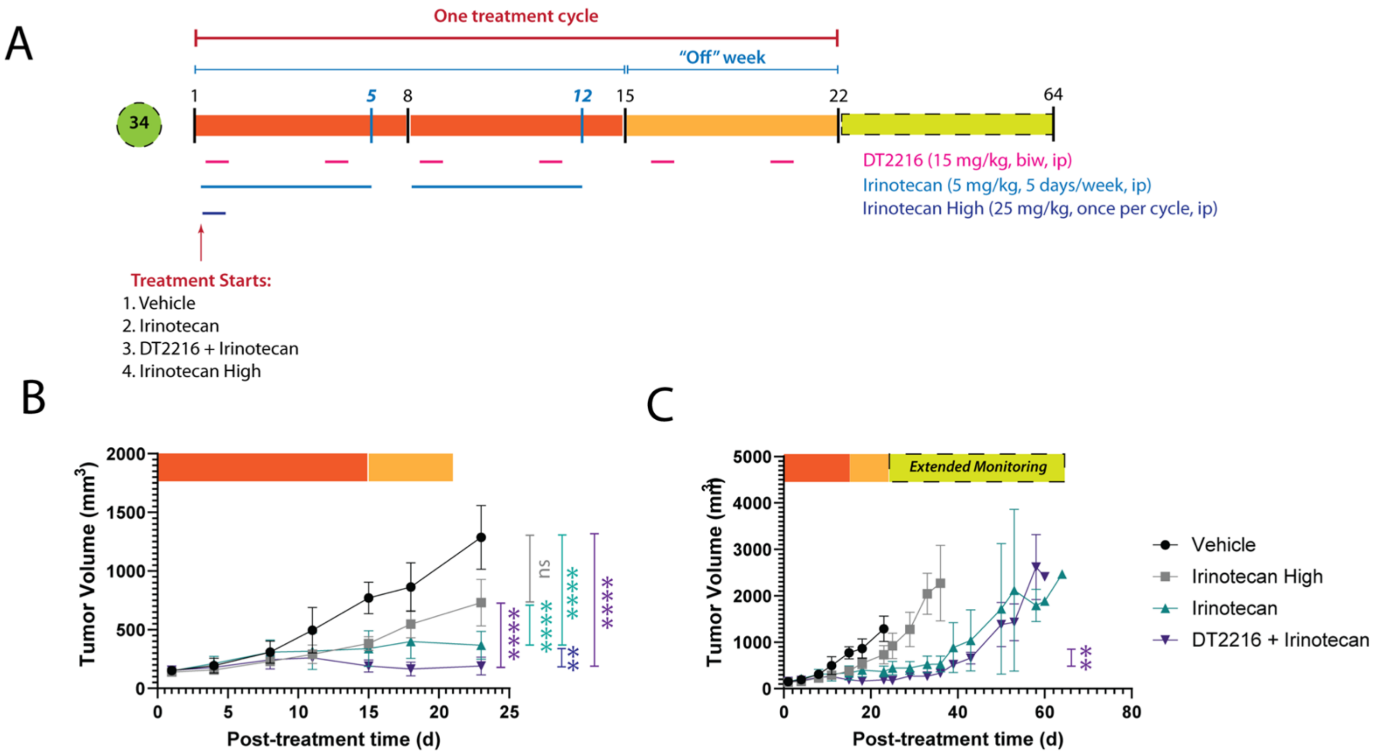

Supplemental Figure 11

Fig. S11: A single high dose of irinotecan is not as efficient as more frequent lower doses of irinotecan. A. Illustration of treatment timeline for PDX 34. DT2216 was administered IP twice a week for the entire treatment cycle, an intermediate dose of irinotecan (5 mg/kg) was administered IP for five days a week for two weeks. A high dose of irinotecan (25 mg/kg) was administered once IP. The third week the mice were off the treatment with low-dose irinotecan. **B.** Changes in tumor volume over the course of the treatment cycle. Data is presented as the mean ± SEM (n = 5 for all treatment groups at the start of the treatment for PDX 34), **C.** Changes in tumor volume over the entire timeline (treatment cycle + extended monitoring beyond treatment). **$p < 0.01$ and ****$p < 0.0001$ in indicated comparisons.
